# Supplementary material for: Genetic heritage of the Baphuthi highlights an over-ethnicized notion of “Bushman” in the Maloti-Drakensberg, southern Africa
Source: Am J Hum Genet. 2023 Apr 26;110(5):880–94. doi: 10.1016/j.ajhg.2023.03.018 (PMC10183465; doi:10.1016/j.ajhg.2023.03.018)
Supplement: Document S1. Figures S1–S9, Notes S1–S3, and supplemental web resources [file mmc1.pdf]

The American Journal of Human Genetics, Volume *110*

Supplemental information

Genetic heritage of the Baphuthi highlights an over-ethnicized notion of “Bushman” in the Maloti-Drakensberg, southern Africa

Ryan Joseph Daniels, Maria Eugenia D'Amato, Mpasi Lesaoana, Mohaimin Kasu, Karen Ehlers, Paballo Abel Chauke, Puseletso Lecheko, Sam Challis, Kirk Rockett, Francesco Montinaro, Miguel González-Santos, and Cristian Capelli

1 Supplemental Notes

Supplemental Note S1: Discussion on the use of population 'labels'

On the backdrop of colonial era racism, slavery, and genocide, people are rightfully sensitive about the words used to discuss them [1-3]. Participants in research should feel respected and that their sentiments are understood both to encourage ongoing public trust and for growth within the scientific community.

Terms used in our paper, such as ‘Hottentot’, ‘Khoisan’, ‘Bantu’ and ‘Bushman’ have a negative connotation to many who have been presently or historically referred to as such [4, 5] . All identifiers or ethnic labels carry some negative connotation because conflict with external communities is unavoidable. As such it is very challenging to find non-offensive terms when referring to communities and moreover when attempting to co-ordinate the use of terms across regions where histories are different. For example, while is South Africa the term ‘Bushman’ is strongly derogatory, some Kalahari hunter-gatherers prefer the name ‘Bossiesmans’ (Afrikaans for ‘Bushmen’) [5].

Despite ongoing debate on the use of ethnic labels, some recommendations are available [6] (Supplemental Web Resource S1). The Working Group of Indigenous Minorities in Southern Africa and the South African San Institute now represent the Indigenous communities in the region. Following the 2003 African Human Genome Initiative conference the two institutions declared a preference for their individual community names or collectively as San. The term Khoe–San was optional when discussing the San and Khoikhoi (more properly spelled as Khoekhoe) as a collective. ‘Khoisan’ was used by anthropologist Leonard Schultze to refer to the pastoralist Khoi and the hunter-gatherer San. As the term persists in linguistics for the broad and diverse language area, we chose to use it in our work (modified as ‘KhoeSan’). We do not assume that those included are linguistically, culturally or genetically homogenous.

The term Bantu means ‘people’ in the Bantu languages. While the term is not offensive across Africa, in Southern Africa the term can be seen as offensive because of its use by the Apartheid regime in South Africa. As such we opted for using the term solely in the linguistic context of referring to the collection of people who speak the Bantu languages, i.e. Bantu speaking communities. We use the following case structure for Bantu speaking community names and languages.
For Nguni communities, the prefix, which indicates noun class and plural/singular, is lower case followed by a capitalised root word (e.g., amaZulu and isiZulu).
For the other Bantu speaking communities, we use a leading capital letter for proper nouns (e.g. Sesotho and Basotho).

Lastly, as our paper attempts to investigate possible genetic distinctions between ‘vanished’ communities who are know mostly, if not entirely, from historic texts, we necessarily need to consider the terminology used in historic texts. Many of these terms have been identified by communities as derogatory and should be avoided [5, 6]. As we do not want to make the field more complicated by introducing new terms, we chose to use the term ‘Bushman’ but solely

to make it clear to which historic references we are discussing.

We are not referring to any contemporary people, participants or communities as ‘Bushmen’ nor do we consider the term ‘Bushman’ to be a legitimate way to refer to people except where it may be the preferred term (e.g. in the Kalahari).
Furthermore, our results show that the notion of a genetically distinct ‘Bushman’ community is unsupported and the term may have no value beyond discussing the use of the word in colonial literature.

Supplemental Note S2: Ethical concerns and data sharing.

Ethical concerns while working with the Baphuthi

Population genetic research necessarily uses information about the genetic identity of participants to discuss the history, present state or future of communities. It is therefore essential that there is a stable channel for communication and understanding between the community and the researchers directly involved in the work to avoid and/or mitigate any possible consequences of the research attention. The public release of genetic and genomic information further adds the need for clear and agreed upon general ethic principles to which researchers, ethics boards and academic journals can adhere and which can be discussed with the participants. With this work we have followed the guidance of the H3Africa working group for genetic research with African communities (Supplemental Web Resource S1). The working group developed a framework for the best practices and an interrogation of the concerns.

In population genetics, the collection of individual results will impact the discussion of and possibly the state of the community to which the individual belongs. The process then of liaising with communities as a collective and/or with community leaders as representatives of the collective is necessary. In our work, communities of interest were approached through Dr Sam Challis and Mr Puseletso Lecheko who have ongoing collaborations with the Baphuthi and neighbouring communities as part of the work of the Rock Art Research Institute. These existing collaborations and the expertise of Dr Challis and Mr Puseletso afforded us much trust with the communities. The community leaders (Chiefs) were informed of the project prior to sampling through in-person visits by our team. The proposed research was explained to the leader and we sought his approval for interviewing people of the community. When verbal approval was granted, we proceeded with the project. The community members who were interested in taking part in the study were provided the opportunity to discuss and question the details of the research with the researchers and with the assistance of a translator.

Data sharing

The data presented in this study was collected for the present study and ongoing work on the history and dynamics of the pre-colonial era for the Southern Bantu-speakers.
Ethics approval and participant written consent allows for the use of the data in future research by researchers not affiliated with the primary data collection team provided that the research abides by the agreement in the original consent forms, ethics application and is deemed appropriate by the primary data collection team. To avoid any misunderstandings, conflicts of interest and violations of the ethics agreement, the request for data sharing will conditioned upon signing a Data Transfer Agreement, supervised by the Technology Transfer Office and the Research Ethics Committee of the UWC, based on the principles stated in the signed Consent Form and information distributed to the participants.
We reserve the right to have the data withdrawn from any work where there has not been adequate discussion prior to the initiation of the project or where the objectives of the project are in conflict with the ethics approval and consent in the original data collection.

Our ethics approval allows for:
- The merging of the current data set with existing or newly generated data.
- The use of existing and/or new data analyses techniques on the data.
- Academic research work which is in a similar vein as the current project, i.e. population genomics, human history, genetic anthropology. We recognise that these are relatively broad areas thus it is necessary for researchers to contact the corresponding authors for data access.
- Further ethics approval applications at the University of the Western Cape, South Africa for work which is deemed to be a meaningful deviation from the original ethics approval and consent.

Our ethics approval does not allow for:
- Any work which directly uses the data for medical, gene – function or evolutionary selection research.
- Any attempt to use the individual genetic profiles to discuss individual phenotypes.
- Any attempt to individually identify the participants based on any of the data (genetic or other).

The provided data will be scrubbed of any identifying information but we further state that any attempt to approach/contact the individual participants based on the provided data for further data collection or to relate other sources of data to individual participants is not permitted.

Supplemental Note S3: Discussion of global PCA results

The first 5 PCs accounted for ~11% of the total variation. Principal component 1 (PC1, explaining ~6% of the variation) separated African from Eurasian individuals (Figure S1, Figure S2). Principal component 2 (~2% of the variation) separated eastern and western Eurasians (FigureS1, Figure S2). Along PC3 (2% of the variation) the KhoeSan individuals are separated from the non-KhoeSan Africans. Principal components 1 and 3 show the Baphuthi from Lesotho and South Africa plot close to the southern Bantu-speaking populations (e.g. amaZulu and Duma San). The Lake Chrissie San are distinctly closer to the Naro, Ju/'hoansi and G|ui and G||ana compared to Duma San and Baphuthi individuals. The recently admixed southern KhoeSan groups (‡Khomani, Nama and Karretjie) are spread toward Eurasian groups along PC1 which is not seen for the Baphuthi nor Lake Chrissie or Duma San.

An east African component found in the horn of Africa (Somali, Oromo, Amhara) is identified by PC4 (Figure S1). On this PC the Baphuthi, Duma San and southern Bantu-speaking groups are shifted toward the KhoeSan groups, away from east Africans. On PC5, west Africans are separated from east and southern Africans and here we see the Baphuthi and Duma San are at the extreme end of the Southern African cline, beyond the other SBE (Figure S1, Figure S2). The Lake Chrissie San are closer to the Hai||om than the Baphuthi and Duma San on PC5.

While accounting for <1% of the variation, PC 7 and 9 highlights two interesting affinities in the Baphuthi. On PC7 the Baphuthi plot at the extreme end near the Southern African KhoeSan, as opposed to the Juu and Khoe-Kwadi at the other end. The Southern African SBE are also shifted in this direction but the Baphuthi are well beyond other South African KhoeSan descendants (Duma San, Lake Chrissie San).
The PC9 distinguishes a western/Juu (e.g. Xun, Ju/'hoansi) KhoeSan component from a southern/Taa (‡Khomani, Karretjie) and Khoe-Kwadi component (Nama etc.) (Figure S1, Figure S2). While the Southern African SBE (including the Duma San) are shifted toward the western/Juu compared to the eastern African SBE, the Baphuthi from Lesotho are shifted further. The Lake Chrissie San, in contrast, are slightly off centre toward the southern/Taa groups.

The outlying position of the Baphuthi_LE along PC5 and 7 is unlikely due to a SNP-chip artefact as we do not see a unique Baphuthi_LE component in the unsupervised ADMIXTURE analyses (Figure S4). This suggests that the outlying position instead reflects possible another evolutionary cause within the Baphuthi related specifically to a reduction in diversity in the KhoeSan (PC9) and southern Bantu-speaker affinities (PC5).

2 Supplemental Figures

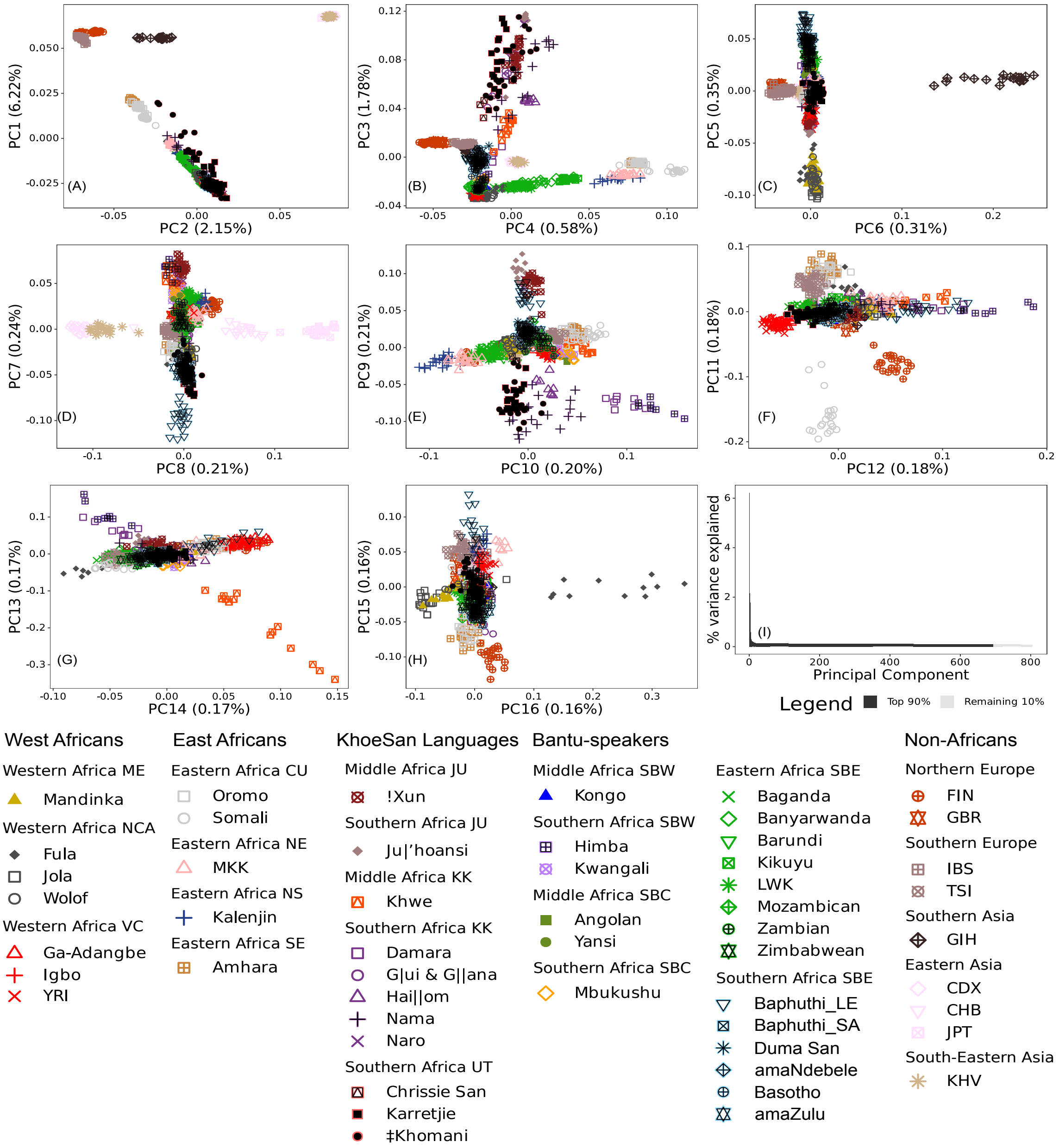

(A)
PC1 (6.22%)
PC2 (2.15%)
(B)
PC3 (1.78%)
PC4 (0.58%)
(C)
PC5 (0.35%)
PC6 (0.31%)
(D)
PC7 (0.24%)
PC8 (0.21%)
(E)
PC9 (0.21%)
PC10 (0.20%)
(F)
PC11 (0.18%)
PC12 (0.18%)
(G)
PC13 (0.17%)
PC14 (0.17%)
(H)
PC15 (0.16%)
PC16 (0.16%)
(I)
% variance explained
Principal Component
Legend
Top 90%
Remaining 10%
West Africans
Western Africa ME
Mandinka
Western Africa NCA
Fula
Jola
Wolof
Western Africa VC
Ga-Adangbe
Igbo
YRI
East Africans
Eastern Africa CU
Oromo
Somali
Eastern Africa NE
MKK
Eastern Africa NS
Kalenjin
Eastern Africa SE
Amhara
KhoeSan Languages
Middle Africa JU
!Xun
Southern Africa JU
Ju|'hoansi
Middle Africa KK
Khwe
Southern Africa KK
Damara
G|ui & G||ana
Hai||om
Nama
Naro
Southern Africa UT
Chrissie San
Karretjie
‡Khomani
Bantu-speakers
Middle Africa SBW
Kongo
Southern Africa SBW
Himba
Kwangali
Middle Africa SBC
Angolan
Yansi
Southern Africa SBC
Mbukushu
Eastern Africa SBE
Baganda
Banyarwanda
Barundi
Kikuyu
LWK
Mozambican
Zambian
Zimbabwean
Southern Africa SBE
Baphuthi_LE
Baphuthi_SA
Duma San
amaNdebele
Basotho
amaZulu
Non-Africans
Northern Europe
FIN
GBR
Southern Europe
IBS
TSI
Southern Asia
GIH
Eastern Asia
CDX
CHB
JPT
South-Eastern Asia
KHV

Figure S1: Principal component analysis of all data. PCA plots in subplots A – H and histogram of percentage variation explained by each component shown in subplot I. Focal populations are indicated in the plots by a black symbol overlaying the coloured symbol. Colours indicate regional and linguistic divisions. Linguistic abbreviations: Mande - ME, North-Central Atlantic - NCA, Volta-Congo - VC, Cushtic – CU, Nilotic eastern - NE, Nilotic southern- NS, Semitic - SE, southern Bantoid western Bantu– SBW, southern Bantoid central western Bantu– SBC, southern Bantoid eastern Bantu– SBE, Juu KhoeSan – JU, Khoe-Kwadi KhoeSan – KK, Ui! and Taa KhoeSan – UT.

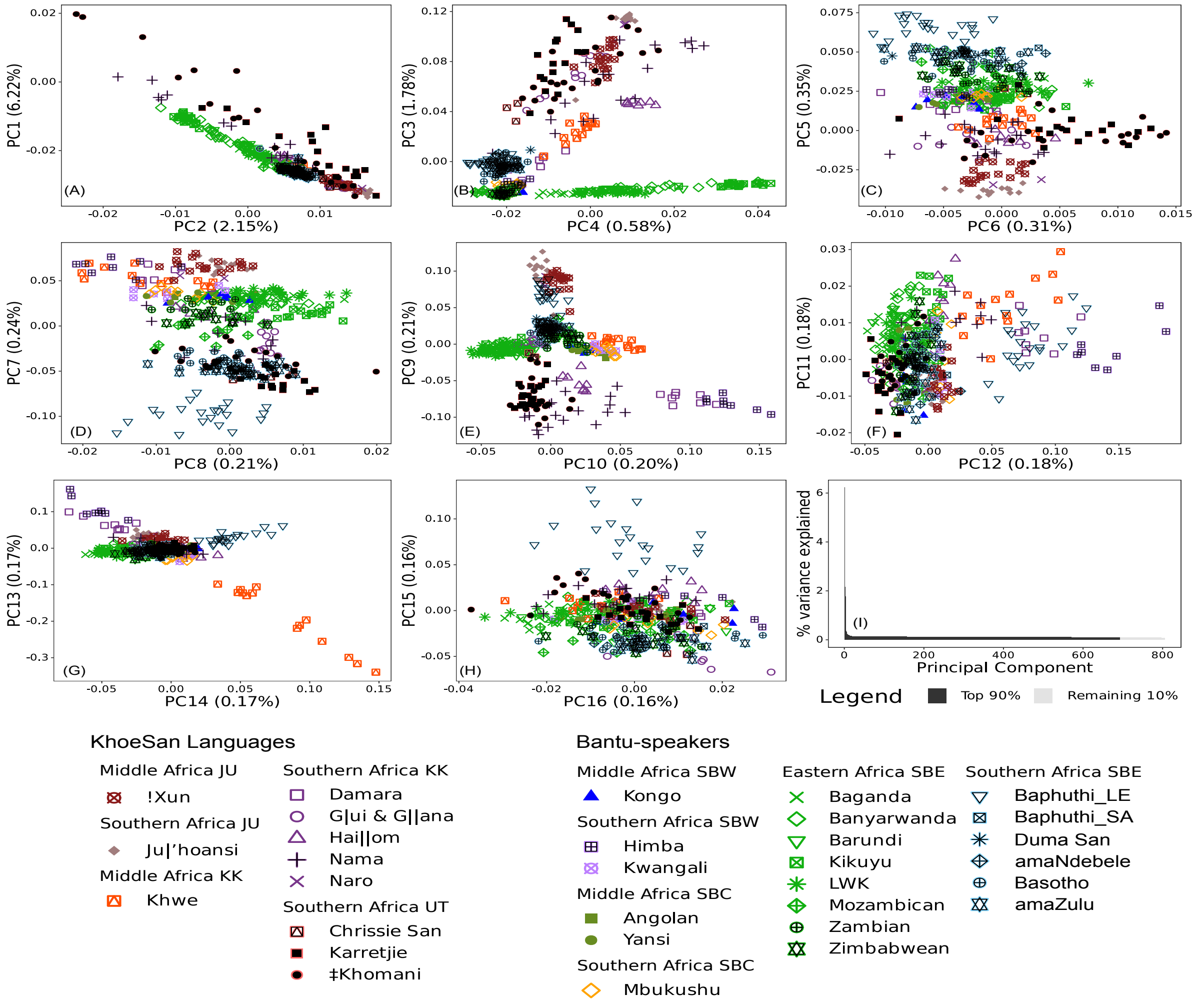

Figure S2: Principal component analysis of Southern African data. PCA plots in subplots A – H and histogram of percentage variation explained by each component shown in subplot I. Focal populations are indicated in the plots by a black symbol overlaying the coloured symbol. Colours indicate regional and linguistic divisions. Linguistic abbreviations: Mande - ME, North-Central Atlantic - NCA, Volta-Congo - VC, Cushtic – CU, Nilotic eastern - NE, Nilotic southern- NS, Semitic - SE, southern Bantoid western Bantu– SBW, southern Bantoid central western Bantu– SBC, southern Bantoid eastern Bantu– SBE, Juu KhoeSan – JU, Khoe-Kwadi KhoeSan – KK, Ui! and Taa KhoeSan – UT.

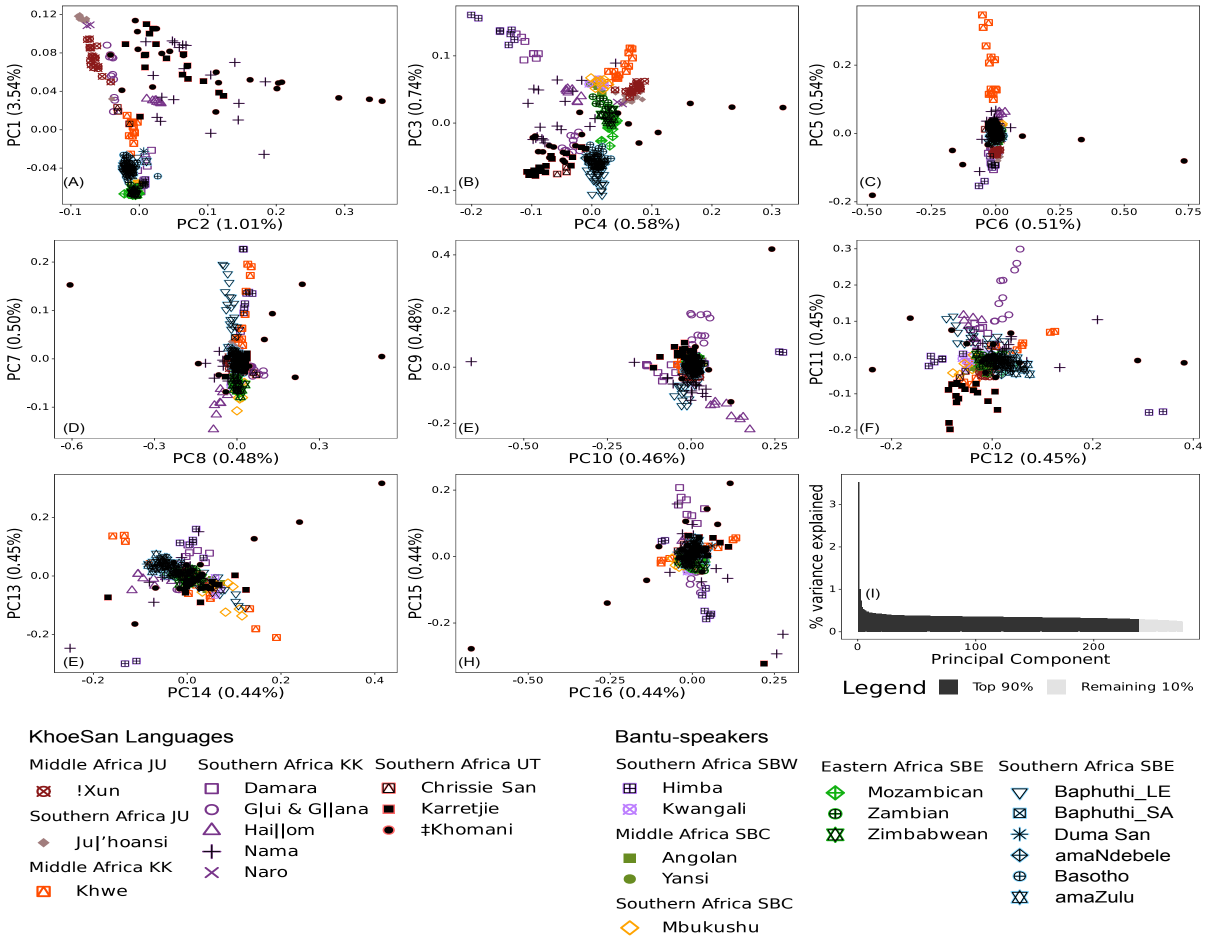

Figure S3: Principal component analysis of all data, plotting only the Southern African samples. PCA plots in subplots A – H and histogram of percentage variation explained by each component shown in subplot I. Focal populations are indicated in the plots by a black symbol overlaying the coloured symbol. Colours indicate regional and linguistic divisions. Linguistic abbreviations: Mande - ME, North-Central Atlantic - NCA, Volta-Congo - VC, Cushtic – CU, Nilotic eastern - NE, Nilotic southern- NS, Semitic - SE, southern Bantoid western Bantu– SBW, southern Bantoid central western Bantu– SBC, southern Bantoid eastern Bantu– SBE, Juu KhoeSan – JU, Khoe-Kwadi KhoeSan – KK, !Ui and Taa KhoeSan – UT.

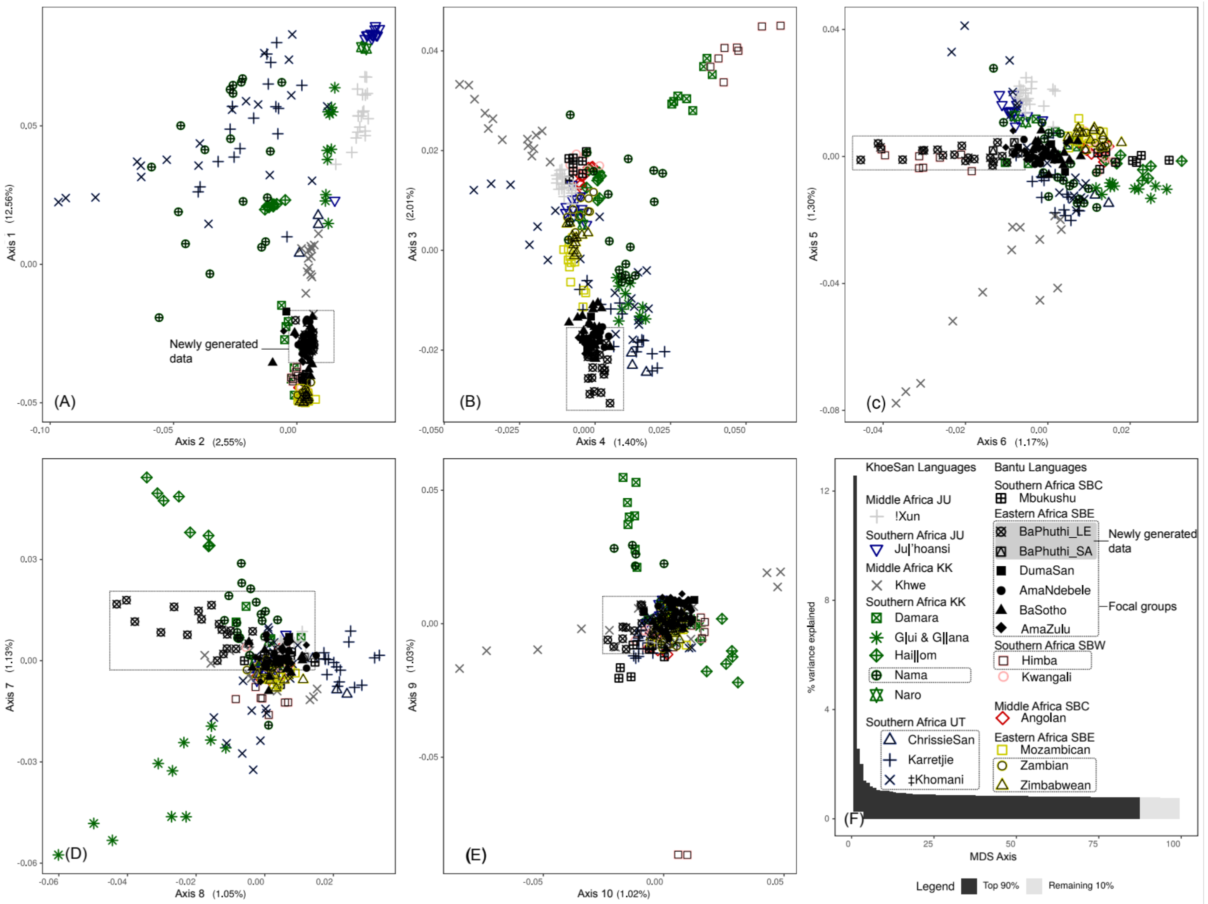

Figure S4: Multidimensional scaling analysis of only the Southern African samples. Focal populations are indicated in the plots by a black symbol overlaying the coloured symbol and by dotted boxes in the figure key. Scaling plots in subplots A – E and histogram of percentage variation explained by each component shown in subplot F. Colours indicate the regional and linguistic divisions. The newly generated data are indicated with a dotted box in both the figure key and the plot. Linguistic abbreviations: Mande - ME, North-Central Atlantic - NCA, Volta-Congo - VC, Cushtic – CU, Nilotic eastern - NE, Nilotic southern- NS, Semitic - SE, southern Bantoid western Bantu– SBW, southern Bantoid central western Bantu– SBC, southern Bantoid eastern Bantu– SBE, Juu KhoeSan – JU, Khoe-Kwadi KhoeSan – KK, !Ui and Taa KhoeSan – UT.

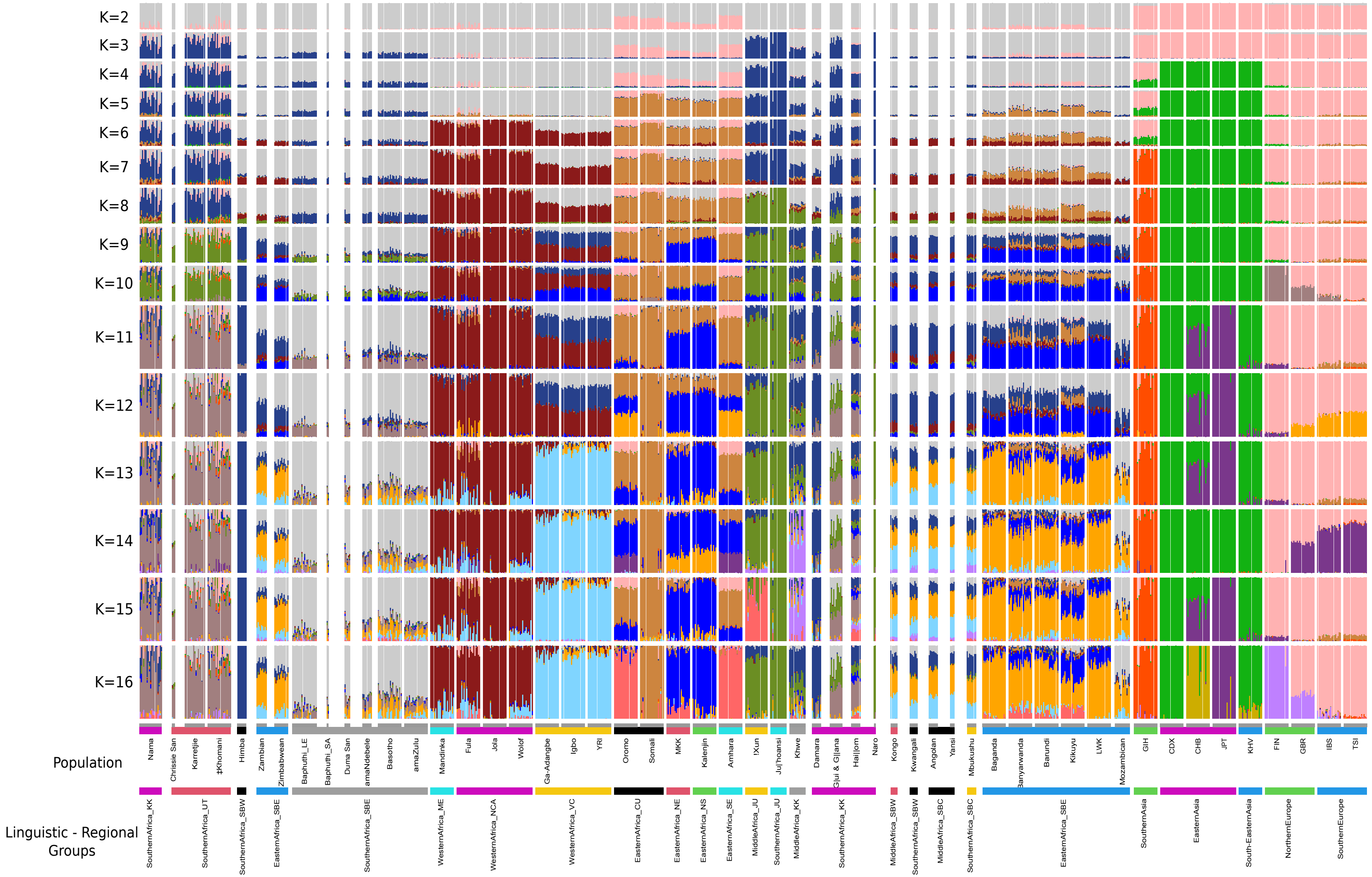
K=2
K=3
K=4
K=5
K=6
K=7
K=8
K=9
K=10
K=11
K=12
K=13
K=14
K=15
K=16
Population
Nama
Chrissie San
Karretjie
‡Khomani
Himba
Zambian
Zimbabwean
Baphuthi_LE
Baphuthi_SA
Duma San
amaNdebele
Basotho
amaZulu
Mandinka
Fula
Jola
Wolof
Ga-Adangbe
Igbo
YRI
Oromo
Somali
MKK
Kalenjin
Amhara
!Xun
Ju|'hoansi
Khwe
Damara
G|ui & G||ana
Hai||om
Naro
Kongo
Kwangali
Angolan
Yansi
Mbukushu
Baganda
Banyarwanda
Barundi
Kikuyu
LWK
Mozambican
GIH
CDX
CHB
JPT
KHV
FIN
GBR
IBS
TSI
Linguistic - Regional Groups
SouthernAfrica_KK
SouthernAfrica_UT
SouthernAfrica_SBW
EasternAfrica_SBE
SouthernAfrica_SBE
WesternAfrica_ME
WesternAfrica_NCA
WesternAfrica_VC
EasternAfrica_CU
EasternAfrica_NE
EasternAfrica_NS
EasternAfrica_SE
MiddleAfrica_JU
SouthernAfrica_JU
MiddleAfrica_KK
SouthernAfrica_KK
MiddleAfrica_SBW
SouthernAfrica_SBW
MiddleAfrica_SBC
SouthernAfrica_SBC
EasternAfrica_SBE
SouthernAsia
EasternAsia
South-EasternAsia
NorthernEurope
SouthernEurope

Figure S5: ADMIXTURE proportions for K=2 ...16 represented as a stacked bar graph for each individual. Each colour represents a component. Samples have been grouped by Linguistic-geographic regions. Population abbreviations are explained in Table S1.

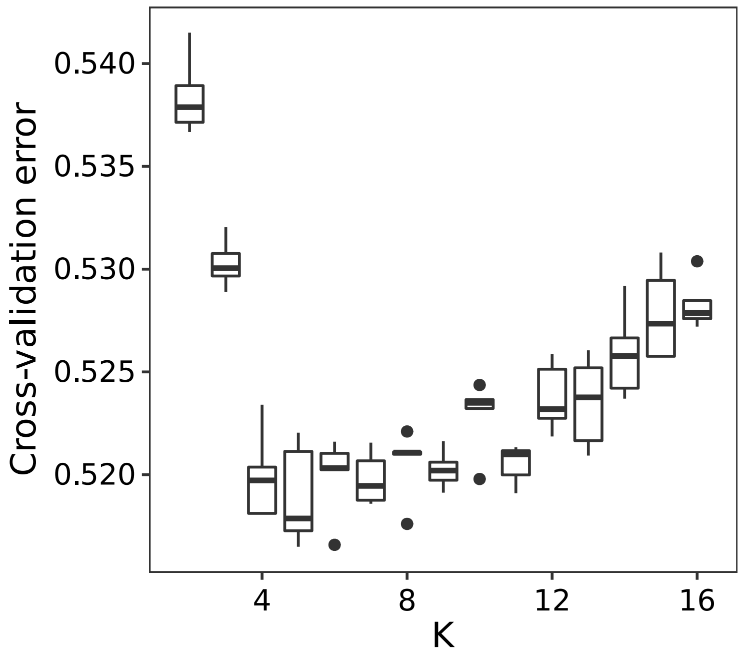

Figure S6: Changes in cross-validation error for K=2..16 with 10 repeated runs each. Dots indicate outlying values. Median value indicated by the line within each box, and the interquartile range is indicated by the extent of the boxes.

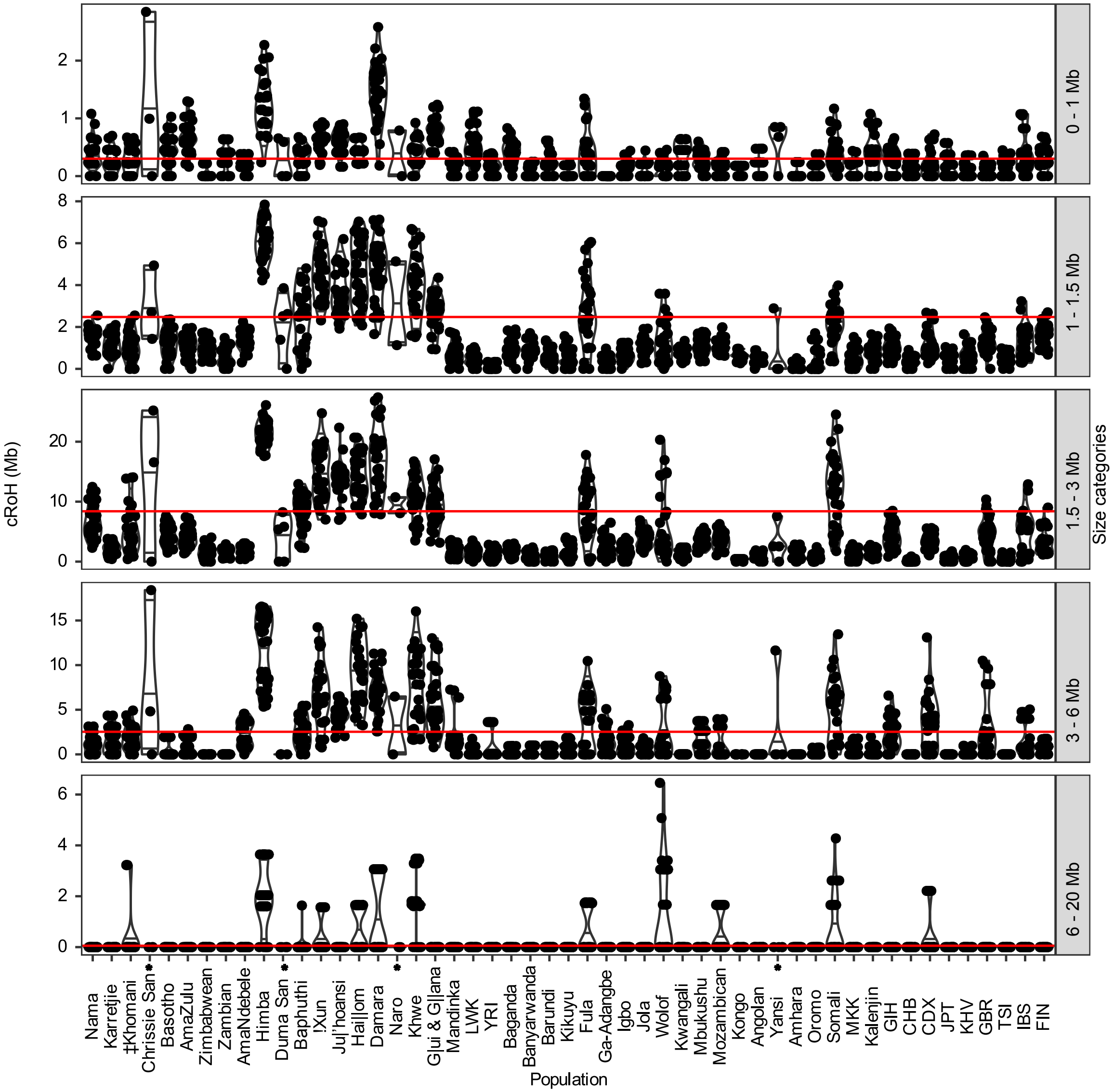
cRoH (Mb)
Size categories
0 - 1 Mb
1 - 1.5 Mb
1.5 - 3 Mb
3 - 6 Mb
6 - 20 Mb
Population
Nama
Karretjie
‡Khomani
Chrissie San*
Basotho
AmaZulu
Zimbabwean
Zambian
AmaNdebele
Himba
Duma San*
Baphuthi
!Xun
Ju|'hoansi
Hai||om
Damara
Naro*
Khwe
G|ui & G||ana
Mandinka
LWK
YRI
Baganda
Banyarwanda
Barundi
Kikuyu
Fula
Ga-Adangbe
Igbo
Jola
Wolof
Kwangali
Mbukushu
Mozambican
Kongo
Angolan
Yansi*
Amhara
Oromo
Somali
MKK
Kalenjin
GIH
CHB
CDX
JPT
KHV
GBR
TSI
IBS
FIN

Figure S7: Cumulative runs of homozygosity (cRoH) for each population across a set of five RoH size bins. Size bins are indicated on the right. Individual dots indicate the average from four individuals in a single iteration (a total of 30 iterations per population). In populations marked with an * we show the estimates for individuals, not iterations, due to small sample sizes. The red line shows the mean of the Baphuthi. Horizontal lines in each violin indicates the 25%, 50% and 75% quantiles.

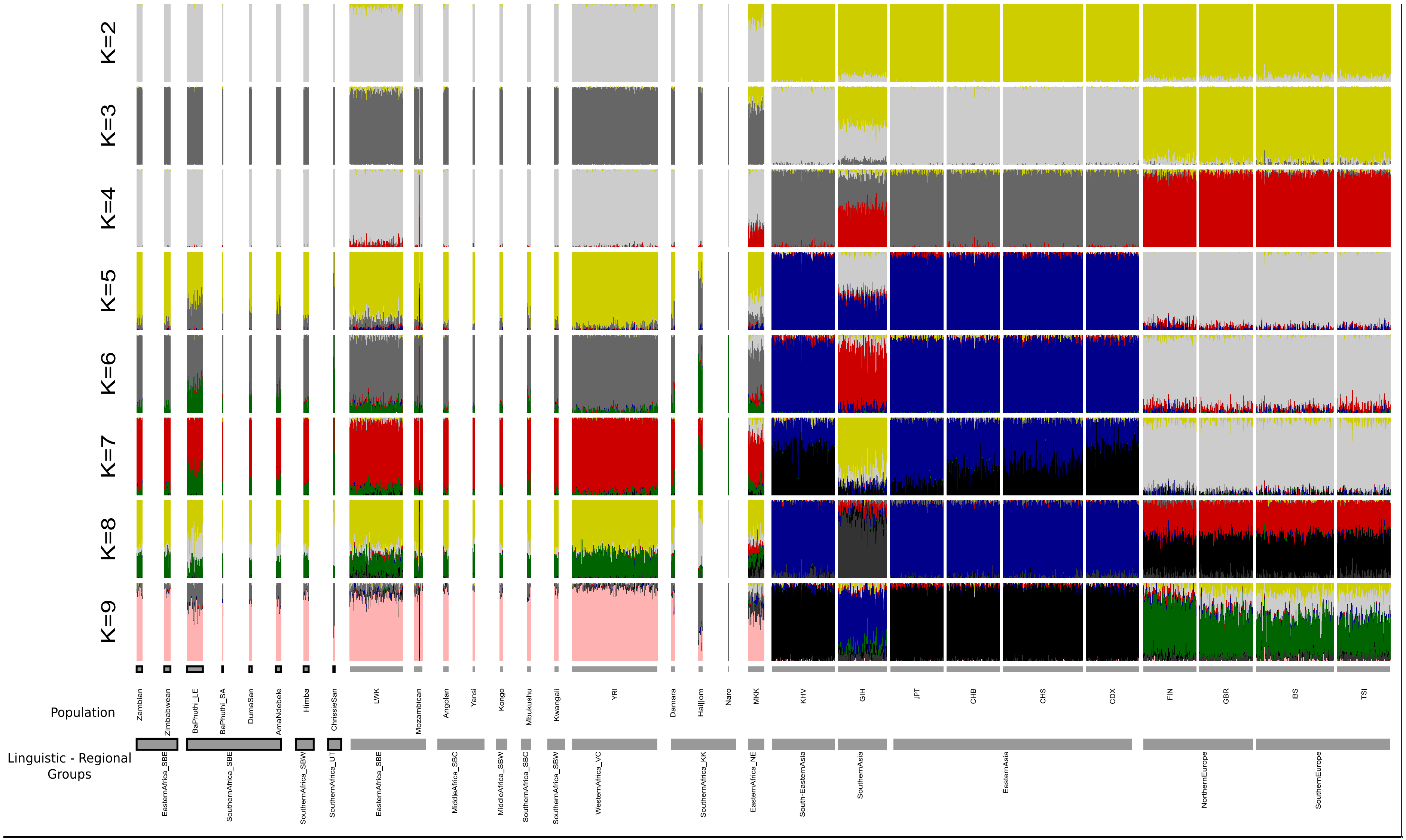

Figure S 8: ADMIXTURE proportions based on the X chromosome data for K=2 ...9 represented as a stacked bar graph for each individual. Each colour represents a component. Samples have been grouped by Linguistic-geographic regions (bars along the x axis). Population abbreviations are explained in Table S1. Focal populations indicated in x axis labels by black border around the bars.

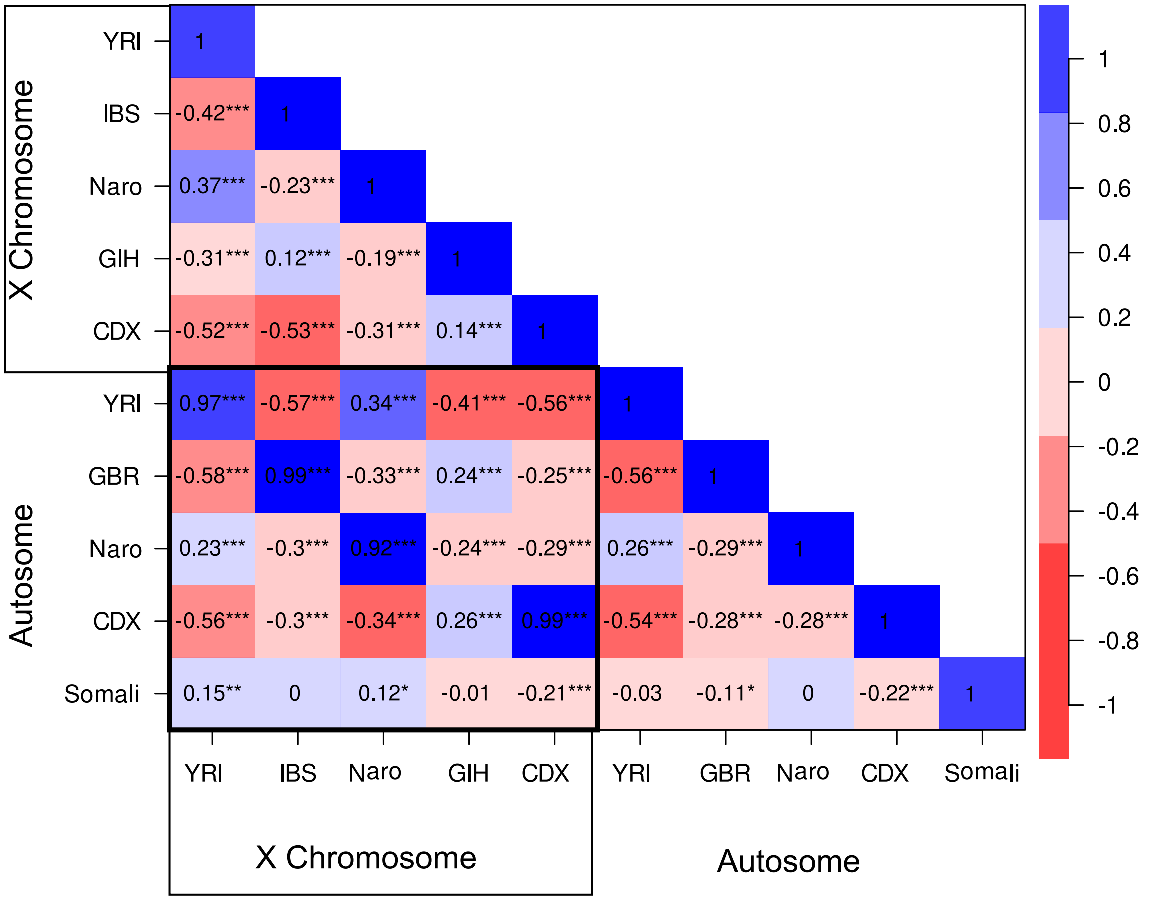

Figure S 9: Pearson's correlation coefficients estimated between pairs of ADMIXTURE components from the autosomal and X chromosome data. Colours in the plot and scale bar correspond to the R-squared value and asterisk indicate level of significance for the p-values. * < 0.05, ** < 0.01, *** < 0.001. Population labels along the x and y axes correspond to the population in which the component was at its greatest, i.e. the proxy source for the ancestry.

2.1 Supplemental Tables

Table S1: List of data included in this paper. Sample sizes after quality control steps (n) are indicated. Language information taken from Glottolog v4 (Web Resource S2). The PubMed ID (PMID) is provided for each data source.
Table S2: Pairwise post-hoc test results for significant differences between populations when comparing ADMIXTURE components at K=9. In the matrix of p-values, the upper right are Nemenyi post-hoc results and the lower left are Baumgartner-Weiß-Schindler results. Statistically significant p-values indicated in *italics*.
Table S3: Estimates of the ADMIXTURE components in each population at K = 9. Indicated are the mean (μ), minimum (Min), maximum (Max) and standard deviation (s.d.) within each population for each component and for selected combinations of components.
Table S4: Results of formal test for admixture using f_3 estimates.
Table S5: Admixture date estimates considering pairs of sources using linkage disequilibrium decay curves. Detected events have been grouped by the language and regions of the identified sources and the date estimates. Where multiple events were detected, the number of events is indicated under 'event'. Symbols : μ – Mean, s.d. – Standard deviation.
Table S6: Estimates of the median ADMIXTURE components in each population for autosomal and X chromosome at K = 5. Indicated are the means within populations for each component (Autosomal and X chromosome) and the ratio of Autosomal:X chromosome for each component. Ratio values were capped at 30 and 1e-06 was added to all components to avoid ratios with a 0 numerator or denominator.

Supplemental Web Resources

Web Resource S1:
H3 Africa Working Group Report 2011. Harnessing Genomic Technologies Toward Improving Health in Africa : Opportunities and Challenges. http://h3africa.org/About/White-Paper
Web Resource S2:
Hammarström, H., Forkel, R. and Haspelmath, M. (2019). Glottolog 4.0. https://glottolog.org/

Supplemental References

1. Berg, K., Bonham, V., Boyer, J., Brody, L., Brooks, L., Collins, F., Guttmacher, A., McEwen, J., Muenke, M., Olson, S. et al. (2005). The Use of Racial, Ethnic, and Ancestral Categories in Human Genetics Research. Am. J. Hum. Genet. *77*, pp. 519-532.
2. Tang, H., Quertermous, T., Rodriguez, B., Kardia, S.L.R., Zhu, X., Brown, A., Pankow, J.S., Province, M.A., Hunt, S.C., Boerwinkle, E. et al. (2005). Genetic Structure, Self-Identified Race/Ethnicity, and Confounding in Case-Control Association Studies. Am. J. Hum. Genet. *76*, pp. 268-275.
3. Morning, A. (2014). And you thought we had moved beyond all that: biological race returns to the social sciences. Ethnic Racial Stud. *37*, pp. 1676-1685.
4. Kent, S. (2002). Interethnic Encounters of the First Kind: An Introduction. In Ethnicity, Hunter-Gatherers, and the "Other": Association or Assimilation in Africa, Kent, S. ed. (Smithsonian Institution Press).
5. Schlebusch, C.M. (2010). Issues raised by use of ethnic-group names in genome study. Nature *464*, pp. 487.

6. Chennells, R. and Steenkamp, A. (2016). International Genomics Research involving the San People. In “Ethics Dumping” – Paradigmatic Case Studies: A report for TRUST, Schroeder, D., Lucas, J. C., Fenet, S. and Hirsch, F. eds. (TRUST Project), pp. 35-40.
