# Supplementary material for: Genetic heritage of the Baphuthi highlights an over-ethnicized notion of “Bushman” in the Maloti-Drakensberg, southern Africa
Source: Am J Hum Genet. 2023 Apr 26;110(5):880–94. doi: 10.1016/j.ajhg.2023.03.018 (PMC10183465; doi:10.1016/j.ajhg.2023.03.018)
Supplement: Document S2. Article plus supplemental information [file mmc3.pdf]

Genetic heritage of the Baphuthi highlights an over-ethnicized notion of "Bushman" in the Maloti-Drakensberg, southern Africa

Authors

Ryan Joseph Daniels, Maria Eugenia D'Amato, Mpasi Lesaoana, ..., Francesco Montinaro, Miguel González-Santos, Cristian Capelli

Correspondence

jryan.daniels@gmail.com (R.J.D.),
cristian.capelli@unipr.it (C.C.)

To safely gain insight into ancient communities from contemporary people, we need to acknowledge that notions of identity change. The genomes of the southern African Baphuthi show that references to KhoeSan antecedents in their history most likely reflect the subjective, inconsistent use of "KhoeSan" terminology and not genetics.

Daniels et al., 2023, The American Journal of Human Genetics *110*, 880–894
May 4, 2023 © 2023 The Authors.
https://doi.org/10.1016/j.ajhg.2023.03.018

CellPress

ARTICLE

Genetic heritage of the Baphuthi highlights an over-ethnicized notion of "Bushman" in the Maloti-Drakensberg, southern Africa

Ryan Joseph Daniels,[1,2,*] Maria Eugenia D'Amato,[2] Mpasi Lesaoana,[2,3] Mohaimin Kasu,[2] Karen Ehlers,[4] Paballo Abel Chauke,[5] Puseletso Lecheko,[6] Sam Challis,[6] Kirk Rockett,[7] Francesco Montinaro,[1,8] Miguel González-Santos,[1] and Cristian Capelli[1,9,*]

Summary

Using contemporary people as proxies for ancient communities is a contentious but necessary practice in anthropology. In southern Africa, the distinction between the Cape KhoeSan and eastern KhoeSan remains unclear, as ethnicity labels have been changed through time and most communities were decimated if not extirpated. The eastern KhoeSan may have had genetic distinctions from neighboring communities who speak Bantu languages and KhoeSan further away; alternatively, the identity may not have been tied to any notion of biology, instead denoting communities with a nomadic "lifeway" distinct from African agro-pastoralism. The Baphuthi of the 1800s in the Maloti-Drakensberg, southern Africa had a substantial KhoeSan constituency and a lifeway of nomadism, cattle raiding, and horticulture. Baphuthi heritage could provide insights into the history of the eastern KhoeSan. We examine genetic affinities of 23 Baphuthi to discern whether the narrative of KhoeSan descent reflects distinct genetic ancestry. Genome-wide SNP data (Illumina GSA) were merged with 52 global populations, for 160,000 SNPs. Genetic analyses show no support for a unique eastern KhoeSan ancestry distinct from other KhoeSan or southern Bantu speakers. The Baphuthi have strong affinities with early-arriving southern Bantu-speaking (Nguni) communities, as the later-arriving non-Nguni show strong evidence of recent African admixture possibly related to late-Iron Age migrations. The references to communities as "San" and "Bushman" in historic literature has often been misconstrued as notions of ethnic/biological distinctions. The terms may have reflected ambiguous references to non-sedentary polities instead, as seems to be the case for the eastern "Bushman" heritage of the Baphuthi.

Introduction

Using contemporary people as proxies for ancient communities is a contentious practice in anthropology[1–5] and an ongoing discussion in trying to understand the relationship between KhoeSan peoples and culture in southern Africa.[3,6,7] As communities and cultures are continually reinvented and lost, only an imperfect account of the past can be gathered from extant cultures and people.[1] Researchers look toward physical remains and historic accounts as well but connecting past descriptions to contemporary peoples may be misleading. Ethnic labels are continually formed, morphed, appropriated, and lost through time. KhoeSan refers to the collective of linguistically and culturally diverse African communities from a range of environments, regions, and times.[5,6] Possible cultural, genetic, and/or linguistic distinctions between eastern and western KhoeSan are incompletely understood.[8,9] The term "KhoeSan" and the many precursor terms are contentious because of their historic use and we refer readers to Note S1 for fuller discussion on the terms used here.

In the western parts of South Africa, KhoeSan have often been referred to as "San," "Hottentots," or "Khoikhoi,"[10,11] while those who inhabited mountainous regions of eastern southern Africa—present-day Lesotho, KwaZulu-Natal, Griqualand East, and the former Transkei—were likely to have all been !Ui San language-speakers[8] and have been referred to as "Bushman" or "Mountain Bushman"[10,12–14] by Europeans and "BaTwa" by the Nguni speakers and "Baroa" by the Sesotho speakers, which are both Bantu-language groups. In terms of language and identity, a few KhoeSan communities have persisted in the western regions, although with notable influences from historic events, including loss of language and other indigenous knowledge, cultural creolization, displacement, and genetic admixture.[3,7,8,15–17] In eastern southern Africa, however, there are few known remnant KhoeSan communities—by name or culture—from which to draw insights.[18]

[1]Department of Zoology, University of Oxford, Oxford, OX1 3SZ Oxfordshire, UK; [2]Forensic DNA Laboratory, Department of Biotechnology, University of the Western Cape, Cape Town 7535, South Africa; [3]Lesotho Mounted Police Service, Technical Support Services, Maseru 100, Lesotho; [4]Department of Genetics, University of the Free State, Bloemfontein 9300, South Africa; [5]Computational Biology Division, Department of Integrative Biomedical Sciences, Institute of Infectious Disease and Molecular Medicine, CIDRI Africa Wellcome Trust Centre, Faculty of Health Sciences, University of Cape Town, Cape Town, South Africa; [6]Rock Art Research Institute, School of Geography, Archaeology and Environmental Studies, University of the Witwatersrand, Johannesburg 2050, South Africa; [7]Wellcome Centre for Human Genomics, Oxford, OX3 7BN Oxfordshire, UK; [8]Department of Biology-Genetics, University of Bari, Via E. Orabona, 4, 70124 Bari, Italy; [9]Dipartimento delle Scienze Chimiche, della Vita e della Sostenibilità Ambientale, Università di Parma, 43121 Parma, Italy
*Correspondence: jryan.daniels@gmail.com (R.J.D.), cristian.capelli@unipr.it (C.C.)
https://doi.org/10.1016/j.ajhg.2023.03.018.
© 2023 The Authors. This is an open access article under the CC BY license (http://creativecommons.org/licenses/by/4.0/).

Any possible ancient signals of divergence are made more complex by historic and ongoing developments. Clear influences are found in cultural diffusion and genetic exchange associated with a number of events. For example, the arrival of the east African pastoralists to southern Africa ~3,000 years ago brought exogenous iron, pottery, genes, and livestock.[3,9,19,20] The subsequent extensive spread of Bantu-language communities (sometimes referred to as Iron Age groups) brought iron-technology with sedentary agro-pastoralism and socio-political change.[9,21–24] The mounting pressure from European colonial expansion and Bantu-speakers' nation-building during the 1600s–1900s decimated, displaced, and in some cases enslaved KhoeSan societies.[25] Such "vanished" communities are known largely, if not entirely, from the records of early travelers and missionaries, as archives such as those of Bleek and Lloyd,[26] as well as their occupational remains and material culture.[27]

What we know of the western KhoeSan was detailed from early encounters with Europeans, which has provided insight into pre-colonial communities.[10,28,29] Historic references to the "San" invoke racialized imagery of smaller stature and paler skin than southern African Khoekhoe and agro-pastoralists:[11,30] early anthropologists describe distinct eye folds, cranial structure, and tight hair curls.[31–33] Linguistic work on contemporary people and from historic accounts have allowed the mapping of possible distributions of linguistically identifiable groups and relationships between San communities in the West. These details are largely lacking for KhoeSan in the East.

Some information on the eastern KhoeSan may be gathered from the ambiguous references to Bushman raiders in the seminal works by Wright[34] and Vinnicombe.[13] Here "Bushmen" are described as akin to the "San" however, the distinction is most likely hyper-ethnicized. As with the division between "San" and non-"San," the "Bushman" reference is rooted in and perpetuated by colonial tendencies to emphasize "essentialist" differences.[35] The connotation of "Bushman" to African and European authorities in the 19th century was pejorative,[35] and terms such as "San," "BaTwa," and "Baroa" most likely denoted a shared "lifeway," not necessarily any notion of "race."[7] Recent work, most notably that of Rachel King and Sam Challis,[7,16,35–37] argues that one could adopt the lifeway and become a "Bushman" and that "Bushman" communities were ethnically heterogeneous, only sharing a lifeway that spurned sedentary polities in favor of hunting, gathering, and livestock raiding.[7,35] Indeed, close relations between KhoeSan and Bantu-speaking communities are a characteristic of the Maloti-Drakensberg history.[35]

The two largest population groups among the Bantu-speakers of southern Africa are the Nguni-speakers and the Sotho-Tswana speakers (web resources).

The boundaries between these (and several other) ethnolinguistic groups are notably obscure, in part because of the recency of their divergences and in part because of migrations, admixtures, and cultural exchanges in the last two centuries.[21,27,29,38]

The linguistic antecedents of these groups migrated southward into southern Africa by the fifteenth century,[8,21–23,39] but the antecedents of the Nguni-speakers may have arrived earlier than that of the Sotho-Tswana communities.[8,40,39]

The contemporary Baphuthi of the southern Maloti-Drakensberg are an interesting community, as they speak Siphuthi, which is a hybrid of these two language groups.[41] From the 1700s, the Baphuthi were ethnically heterogeneous.[11,39,41–44] While the Baphuthi's history is rooted in the amalgamation of southern Bantu-speaking communities including Nguni-speaking (such as the amaZizi and the Mpondomise) and Sesotho-speaking (e.g., Maphuthing, Bafokeng),[43] their oral history and identity attests to KhoeSan heritage.[43] The Baphuthi rejected sedentary "Great Place" chieftaincy in favor of circulating through a series of settlements atop steep-sided hills scattered along the Senqu river.[11] This lifeway based on nomadism and cattle raiding, rather than agriculture, was shared with their KhoeSan antecedents and contemporaries.[43] The Baphuthi constituency, which has been historically referred to as "Bushman," were ethnically heterogeneous too, but many of their members were KhoeSan.[34,41,43] Furthermore, the assimilation of some Eastern Cape amaTola, who are yet another KhoeSan-Bantu speaking creolized community,[43] would have added ancestry from KhoeSan speakers, in addition to contributions from escaped slaves and outlawed Europeans.[7,35,36] This recent assimilation of "Bushmen" and amaTola may be reflected as elevated KhoeSan ancestry, as seen in Lake Chrissie communities,[8] but it is unclear to what extent this would be the case for the Baphuthi as the Baphuthi and "Bushmen" of the 1800s are now recognized as ethnically heterogeneous.[25,35,37] We ask whether historic references to "Bushman" heritage does reflect KhoeSan ancestry and whether it can provide insight for an eastern KhoeSan ancestry.

We focus on two contending views for the "Bushman" heritage but acknowledge that neither is exclusive of the other. Firstly, "Bushman" descent may reflect a KhoeSan community with genetic distinctions from the western KhoeSan. Secondly, the "Bushman" ancestry may reflect the assimilation of heterogeneous societies with a shared lifeway but with very limited or no KhoeSan-type genetic affinities.

Efforts toward understanding the genetic diversity of southern African KhoeSan and the history of the region increasingly require the insights from remnant genetic signals in descendant communities, such as the Baphuthi. To this end, we examine the genetic affinities of Baphuthi individuals with oral history of KhoeSan descent from the southern Maloti-Drakensberg.

Subjects and methods

Ethics approval

Ethics approval for the South African samples was obtained from Oxford Tropical Research Ethics Committee (The University of

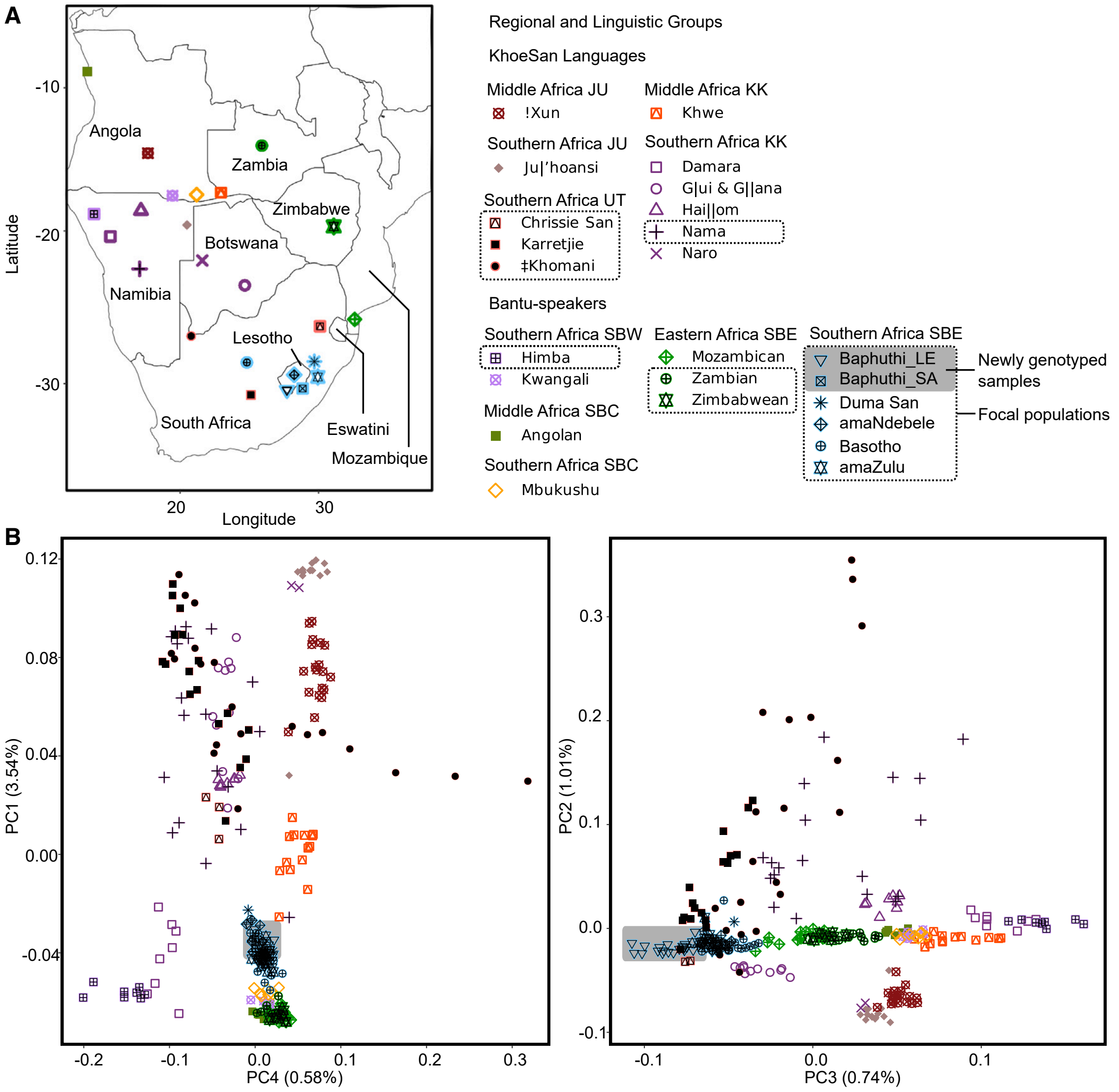

Figure 1. Summary of the geographic and genetic relationship among the Bantu-speaking and KhoeSan populations included in the analyses

(A) A map of the included data. Country labels included.

(B) Principal-component (PC) analysis showing the first four PCs arranged to emphasize patterns. The percentage variation explained by each component is indicated in brackets along the respective axis. The plot symbol colors indicate regional and linguistic divisions. The focal populations are highlighted in the figure key by a dotted box and in the plots by a black symbol overlaying the colored symbol. The newly genotyped data are highlighted with a gray box in the figure key and in the plots. Linguistic abbreviations used: southern Bantoid, western Bantu (SBW), southern Bantoid, Central western Bantu (SBC), southern Bantoid, eastern Bantu (SBE), Juu KhoeSan (JU), Khoe-Kwadi KhoeSan (KK), !Ui and Taa KhoeSan (UT).

Oxford, UK) (ref. no. 8–16) and the University of the Free State (South Africa) NatAgri Ethics Committee (ref. no. UFS-HSD2016/1210). Approval for the Lesotho samples was granted by the University of the Western Cape Research Ethics Committee (ref. no. BM16/3/18) and the Ministry of Health, Lesotho (ID128-20016). Export permits were approved by the South African Department of Health and the Lesotho Ministry of Health. Further details can be found in Note S2.

Sample collection and genotyping

We conducted interviews with residents from Masakala in South Africa (2017) and from Semonkong village and Quthing district in Lesotho (2019) (Figure 1). Information on mother-tongue language, place of birth, and ethnicity of the participant, their parents, and grandparents were collected. Approximately 2 mL of saliva was collected with Oragene-500 kits (DNA Genotek, Canada). To filter the data for individuals who may retain genetic signatures from the historic Baphuthi admixtures (with less influence from very recent admixtures), we considered only participants who indicated that all four grandparents were Baphuthi or spoke Siphuthi for genotyping. DNA extractions were performed at the University of Oxford following the prepIT.L2P salt extraction protocol (catalog# PT-L2P, DNA Genotek, Ottawa Canada). A total of 33 samples from Lesotho were genotyped for over 600,000 SNPs on the Global Screening Array (GSA v2) at the Estonian Institute for Genomics, Tartu, Estonia. A further two samples from South Africa were genotyped for 2.5 million variants on the Illumina

Omni2.5-8 Beadchip v1.3 at the Wellcome Trust Centre for Human Genomics, Oxford University. All raw output was processed with GenomeStudio software (Illumina, USA) and all samples passed a call rate of 97% or more. The Baphuthi population is likely to total only a few thousand, but we could not find census data. This sample set may be representative of the local communities sampled but would not capture the overall regional variation. The sample sizes are however in line with that used in other human admixture studies (see[8,9,22,30]).

Datasets, merging, and quality control

We merged the samples genotyped here with publicly available data genotyped on the Illumina Omni5 or 2.5 array from the African Genome Variation Project,[45] the 1000 Genomes Project,[46] and four southern African datasets.[8,17,19,47] The latter included a collection of 11 KhoeSan groups including representatives of the Khoe-Kwadi (abbreviated to KK), Juu (abbreviated to JU), and !Ui - Taa (abbreviated to UT) language areas (Figure 1A). In all populations except the Baphuthi, we restricted sample sizes to 20 randomly selected individuals to reduce computation load.

The final dataset included a collection of southern African populations to which we pay particular attention to compare with the Baphuthi. These groups are hereafter referred to as the "focal groups." We included several southern African KhoeSan groups: Karretjie, ‡Khomani, and Namibian Nama—as the only representative of Khoekhoe groups. We included several groups from the southern Bantu language communities (here abbreviated to SB). From the southern Bantoid, eastern Bantu language speaking communities (abbreviated as SBE) we included individuals from Zambia, Zimbabwe (the eastern African SBE [web resources]) and from the Basotho, amaZulu, and amaNdebele (southern SBE). The Himba of Namibia were included as a southern Bantoid, western Bantu language community (abbreviated to SBW). We further included two groups of present-day Bantu speakers who historically had KhoeSan affinities: the Lake Chrissie San on the border of Eswatini and the Duma San from the KwaZulu-Natal uKhahlamba-Drakensberg. Both are geographically and linguistically close to the Baphuthi, however based on previous work,[8] we considered the Lake Chrissie San among the KhoeSan and the Duma San among the SBE. The remaining samples made up a "global reference" set from which we infer affinities. Table S1 provides an overview of the grouping.

From each dataset, the following analyses were conducted in PLINK 2.0.[48] We retain only bi-allelic variants and pruned for T/A or C/G polymorphisms to prevent strand ambiguities. We removed SNPs with no chromosomal position and updated coordinates from rsIDs to a custom Chr_position[b37] ID to ensure a match across datasets. The minimum allele frequency was set to 1% (–maf 0.01) and missing genotypes were trimmed per individual and per locus to a maximum 5% (–geno 0.05 –mind 0.05). Second degree and closer relatives (kinship coefficient > 0.087) were removed from all groups with the -king-cutoff 0.088 flag. Outliers were detected iteratively with smartpca from the software package Eigensoft.[49] We based removal on the first five eigenvectors by using five iterations with a sigma threshold of 6 as in Novembre et al.[50] The final dataset comprised 164,100 SNPs, 52 populations, and 806 individuals, of which 23 were Baphuthi ($n = 2$ South Africa, $n = 21$ Lesotho) (Table S1).

Data clustering and population structure

We investigated genetic clustering by using ADMIXTURE v1.3.0[51] and principal-component analysis (PCA). These approaches allowed us to confirm the absence of batch/chip effects or other merging artifacts and to compare the observed population structure to that previously reported. As neither analyses accounts for correlation between SNPs, we trimmed SNPs in linkage disequilibrium (LD).[52] We removed a locus from pairs with $R^2 > 0.7$ for a 50 bp frame with a 5 bp sliding window (PLINK –indep-pairwise 50 5 0.7) as per Busby et al.[23] The SNP count was reduced to 117,358 in these analyses. We further used a multidimensional scaling plot as a third method to examine possible batch effects (PLINK –cluster –mds 10).

The PCA was performed with plink (–pca) both on the entire dataset and with a focus on relevant data from populations south of the African Forest Belt (~5.6 S latitude).

We ran the ADMIXTURE analysis for the autosomal data for K values between 2 and 16, where K is the number of tested clusters. We used ten replicates for each K and a 5-fold cross-validation error (CV) estimation with 100 bootstraps for standard errors (-B100 –cv INPUTFILE.bed {2..16}). The lowest CV error determined the optimum K values.[51] To identify common modes across replicates, we processed the output with the CLUMPAK server[53] by using default settings (LargeKGreedy algorithm, 2,000 random permutations). Results were visualized with ggplot2[54] in R v.3.5.1 (web resources).

We tested for significant differences in each ADMIXTURE component between the focal populations by using a Kruskal-Wallis rank-sum test as implemented in R (kruskal.test) and we identified pairwise differences with two post-hoc tests: the Baumgartner-Weiß-Schindler (bwsAllPairsTest) and the more conservative Nemenyi test (kwAllPairsNemenyiTest), both from R package PMCMRplus and both with a Holms adjustment for multiple corrections (web resources).

Identifying parent populations and admixture dates

As a formal test for admixture in the history of the focal populations, we estimated the f_3 indices[55] in the form of f_3 (X,Y; test population), where X and Y are potential source populations. Focal and reference populations were all included as possible sources. Negative f_3 values (Z score < -3) are considered indicative of a discordant tree relationship and in support of admixture.[56] Estimates were made with Admixtools v.5.1.[57] The Lake Chrissie San were excluded from this analysis, as they were represented by three individuals only.

We estimated the timing of admixture events by fitting exponential decay curves of LD against increasing distances between SNP pairs, as implemented in MALDER.[19,58] Because of sample size, the Lake Chrissie San were excluded. We used an inter-generation time of 28 years, in line with other work.[9,23,59] Events were estimated from 1960 CE, the mean date of birth of the Baphuthi participants. We estimated standard errors by jack-knifing over chromosomes and estimated a Z score by dividing the mean by the standard error.

To understand whether historic and/or ancient bottlenecks and inbreeding have been influential in shaping the Baphuthi genome, we estimated the cumulative size of the genome that has runs of homozygosity (RoH). We followed the procedure of Schlebusch et al.[17] by using PLINK. We subsampled four individuals from each population for a total of 30 iterations to estimate the average cumulative RoH (cRoH) for each of five size categories in Mb; (0;1), [1;1.5), [1.5;3.), [3;6), [6;20). For populations with insufficient sample sizes for subsetting, we plotted estimates for each individual.

Sex biases in admixture based on the X chromosome

To explore in possibility of sex biases in admixture history, we analyzed the X chromosome data by using ADMIXTURE. Data were prepared as described for the autosome but the following adjustments were included as per Ongaro et al.[60]

As X chromosome data were not available for all populations, the dataset was reduced to 27 populations and 3,731 loci after LD trimming. We revised sex classifications by imputing with the genotype data in PLINK (–impute-sex). A male call was made when the rate of homozygosity was >80% and any individuals with ambiguous imputations were removed.

In ADMIXTURE, we set heterozygous SNPs in the male X chromosome as missing and used the option "–haploid = 'male:23" to treat male individuals as haploid. As a result of the filtering, one individual from the Baphuthi_LE was removed.

We summarized the results at K = 5 following the same procedure as for the autosomal data and to facilitate comparisons with the autosome, we also summarized autosomal results. To ensure that the components identified for autosomal and X chromosome were indeed capturing the same regional ancestries and that a comparison would be valid, we performed a Pearson's correlation of components across the two datasets by using the corrplot (web resources) package in R.

Lastly, we focus on the ratio of non-KhoeSan African to KhoeSan ancestry (NKS:KS) as a marker of differentiation among populations in the history of admixture related to the expansion of agro-pastoralism.

The ratio of $(NKS:KS)_{autosomal}$ to $(NKS:KS)_{X\ chromosome}$ then gives an idea of sex biases and variation among populations. A value of 1 indicates no change in the NKS:KS ratio and thus no sex-biased admixture. Low values indicate higher KhoeSan ancestry on the autosome (possible male KhoeSan bias) and high values indicate lower KhoeSan ancestry on the autosome (possible KhoeSan female bias).

Results

Data clustering and population structure

We explored global population structure by using PCA and ADMIXTURE analysis. The patterns observed in the global PCA correspond well with published results on global diversity (Note S3, Figures S1 and S2). When focusing on the PCA among southern African groups (Figure 1B), the first five PCs accounted for ~6.5% of the total variation (Figure S3).

We see that on PCs 1 and 4, all the focal SBE populations—which includes the Baphuthi—cluster close to one another. These two axes accounted for the separation of KhoeSan from non-KhoeSan Africans and western from eastern populations, respectively, and may suggest shared KhoeSan and Bantu-speaking genetic affinities in the focal SBE. The Lake Chrissie San are distinctly closer to KhoeSan groups, in particular the Taa and Khoe-Kwadi groups. The Baphuthi are at the extreme end of PC 3, which captures a gradient between northerly and southerly Bantu-speaking populations (Figure 1). While this might be caused by a batch artifact, we do not see the same separation on other PCs (Figure S3), nor do we see an ADMIXTURE component that is unique to the Baphuthi in the unsupervised analyses (results below). The results of the multidimensional scaling plot show no clear sign that the Baphuthi data are influenced by a batch effect either (Figure S4). On the basis of this, we suggest that the separation on PC 3 reflects instead a Baphuthi-specific change affecting their affinities to other SBE speakers. On PC 2, which seems to account for non-African ancestry in the KhoeSan groups, the Baphuthi are no different from the other focal SBE. In contrast, KhoeSan groups with known Eurasian admixture (‡Khomani, Nama, and Karretjie) separated from the remaining Africans. On PCs 2 and 3, the Chrissie San are shifted more toward the KhoeSan who have little recent Eurasian admixture (e.g., G|ui and G||ana, Hai||om) than the southern African ‡Khomani, Nama, and Karretjie, but they are also shifted toward the southern African SBE groups, showing some genetic similarity to them.

The ADMIXTURE analysis of the global dataset paralleled the variation captured by the PCA described above, in line with previously published work (e.g., Rosenberg et al.[61] and Bryc et al.[62]) reflecting divisions between global regions (Figures 2 and S5). Here we discuss the results for K = 9 (Figure 2), as the cross-validation errors were similar to the lowest estimates (Figure S6). To simplify discussing the ADMIXTURE components, we focus on those present in the focal populations and refer to them on the basis of the populations in which the component is at the highest proportion on average.

The South African and Lesotho Baphuthi had similar ADMIXTURE profiles. On the basis of this and their shared position on all PCA axes, we merged the data and from here forward we discuss the joint Baphuthi data. Significant variation in ADMIXTURE components was found across our focal populations (Baphuthi, Duma San, Lake Chrissie San, and the SBE) (Table S2, Kruskal-Wallis test KW = 106.40, $p < 0.001$). There was some disagreement between post-hoc tests for pairwise differences, but these reflect different levels of conservativeness between the tests. The overall agreement is discussed below.

The Baphuthi profile was composed predominantly of two components but had noteworthy contributions from an additional five components (Figure 2). The most predominant component in the Baphuthi was prevalent across the southern African SBE but was highest in the Baphuthi (mean ~81%, gray in Figure 2). This component distinguished the Bantu language communities from other Africans. The tests for significant differences among focal groups found that the southern SBE had significantly greater proportions of this component compared to all other groups (Figure 3, Tables S2 and S3, post-hoc tests).

The second largest component in the Baphuthi (mean 15% and 12% the lowest seen in any individual; dark green in Figure 2) was strongly related to the KhoeSan groups and was at its greatest in the Naro KhoeSan (98%, hereafter the "Naro" component). In the Baphuthi ADMIXTURE profile, the sum of the "Naro" and "Baphuthi" components (95% ± 4%) was notably greater than the sum of these

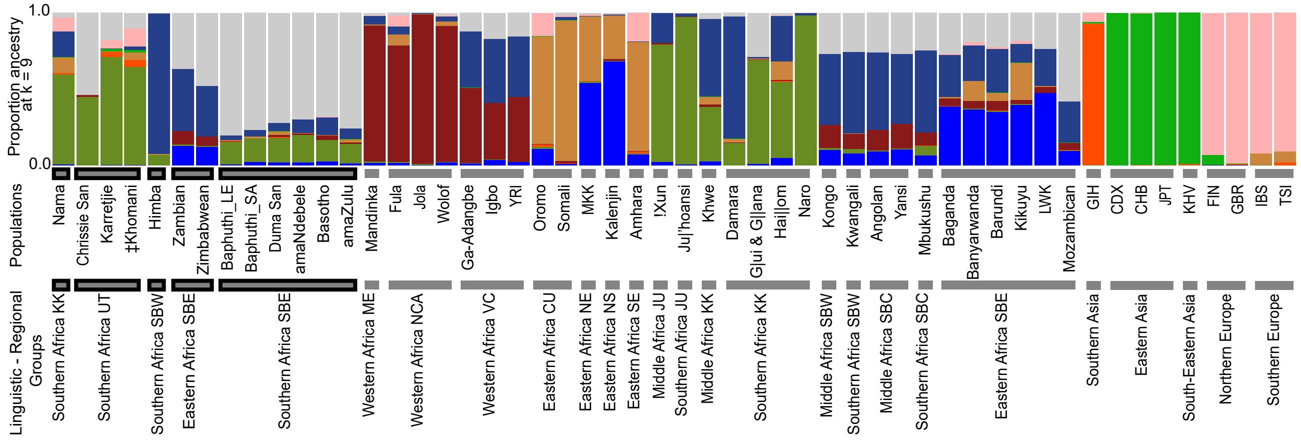

Figure 2. Population-averaged ADMIXTURE proportions for K = 9 represented as stacked bar graphs
Each color represents a component. Samples are plotted in regional-linguistic groups. Population abbreviations are explained in Table S1. Focal populations indicated in x axis labels by black border around the bars.

two components in any other southern SBE (<90%) or eastern SBE (<60%) (Table S3). For example, of the 22 Lesotho Baphuthi, 22% had a sum of more than 99%. Only some KhoeSan groups had individuals with such profiles.

If we look beyond our focal groups, we see that the Naro, Ju'|hoan, and G|ui and G||ana had the sum of these two components comparable to the Baphuthi (>95%). However, in these populations the sum was largely driven by elevated "Naro" proportions (the ratio "Baphuthi":"Naro" was well below 1). The ratio of the two components in the Baphuthi is very similar to the ratio in other southern SBE (median ~5.5 ± 1, Table S3) despite the variation in absolute values. In contrast the ratios of the southern SBE were far greater than the eastern African SBE where the "Naro" component was virtually absent (1 ± 1%; mean ± SD Table S3).

A further distinction of the Baphuthi from the other southern SBE was that they had notably lower proportions of the non-major components. These components likely reflect variation in population history and may be important for understanding divergences between populations.

Of these minor components, the most variable among the southern SBE focal groups was the component dominant in the Himba ("Himba" component, dark blue in Figure 2). Populations from our region of interest, southern Africa (SBE-speaking and UT-speaking communities) had lower proportion of the "Himba" component compared to Bantu-speaking, the KK-speaking, and JU-speaking KhoeSan from other regions (Table S3). The Baphuthi and amaZulu in particular had significantly lower proportions compare to the Basotho, Duma San, and amaNdebele and much lower than the Zambians (41% ± 3%) and Zimbabweans from further north (33% ± 3% Table S2). The Nguni-speakers (amaZulu, amaNdebele, and Duma San) are discrepant in the levels of Himba component. Admixture or drift may have differentiated the Baphuthi and amaZulu from the other southern SBE. There is thus important variation of this western African component in the region. The co-occurrence in relatively similar amounts of the "Baphuthi," "Naro," and "Himba" components are suggestive of a shared history for the southern SBE populations.

The Nama (KK group) are an exception within the region. They have distinctly elevated "Himba" (17% ± 17% Figure 3) and "Somali" (10% ± 5% Figure 3) components, which are comparable to the other Khoe-Kwadi groups. Furthermore, the Baphuthi, Duma San, and Lake Chrissie San can be distinguished from the Nama, Karretjie, and ‡Khomani by the lower levels of Eurasian components (light green and pink in Figure 2).

Identifying parent populations and admixture dates

The overall similarity of the southern SBE was further supported by f_3 admixture tests. For all the southern African SBE (which includes the Baphuthi), when the possible pairs of source populations were set as the KhoeSan-speaking Ju'|hoansi and the west Africans (Yoruba or Igbo from Nigeria), the lowest Z scores ($Z < -10$) were reported, indicating significant support for admixture. When testing for admixture in the eastern SBE, Z scores recovered were lower, for the Zambians, below −8, and the Zimbabweans were not statistically significant ($Z > -3$) (Table S4). Failure to detect the admixture in the Zimbabweans may reflect the lower proportion of KhoeSan ancestry as seen in the low "Naro" ADMIXTURE component.

Another common admixture signal was that of the east African pastoralist gene flow, which we detected in the Nguni-speaking Duma San, amaZulu, amaNdebele, and the Sesotho-speaking Basotho (Z scores < -3) and for all the southern African KhoeSan when we considered source pairs involving non-Bantu east Africans and KhoeSan populations (Table S2)

The signal, however, appears weaker if not absent, in the Baphuthi as well as the east African Zambians and Zimbabweans as no admixture signals were detected.

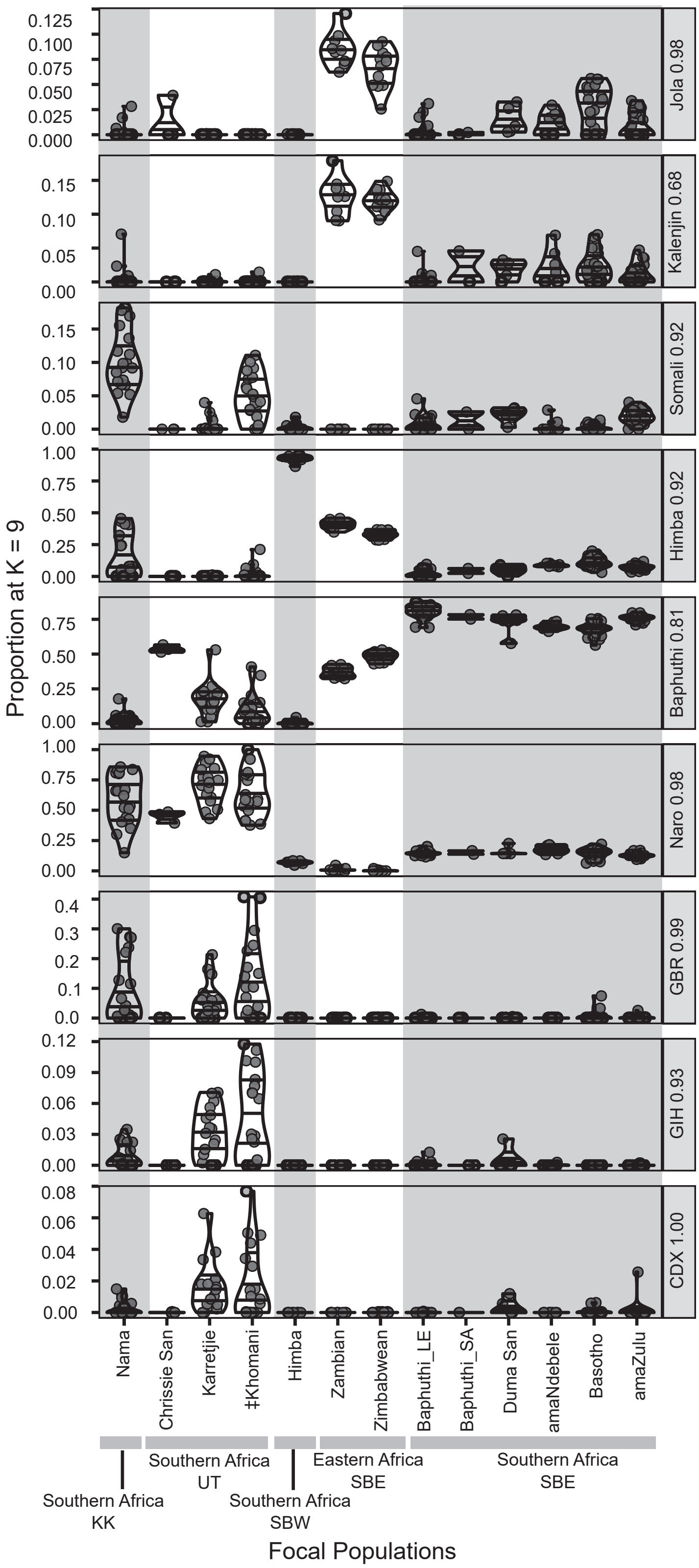

Figure 3. Variation in ADMIXTURE proportions for the focal populations at K = 9
Dots represent individual data points and violin boxes show the range. Horizontal lines within the violins show median and interquartile ranges. Facet labels to the right of each row give the global reference population in which the component is at the highest proportion and the average value within that population. Note that the y axis range varies by facet.

Finally, only for Zambians and Basotho there is evidence of an admixture event between two Bantu-speaking communities. A central southern Bantu (SBC) or SBW admixture with an SBE group is detected in the Zambians. An eastern Africa SBE-southern African Nguni admixture is detected in the Basotho.

As admixture of the SBE with the autochthonous KhoeSan may not have been uniform across the region, we performed a series of tests to distinguish the introgression of KhoeSan contributions that were distinct from that already present in the southern SBE by using f_3 (KhoeSan, South African Bantu-speaking group, target population). The test did not support additional KhoeSan contributions to the Baphuthi, amaZulu, Zimbabweans, or Zambians compared to what is already present in the southern SBE (Table S2). Significant results were detected for the Basotho, Duma San, and amaNdebele. We point out that the Nguni-speakers (amaZulu, amaNdebele, and Duma San) do not show a coherent signal. All the southern KhoeSan produced support for admixture with these pairs of sources, indicating that KhoeSan-related proportions and/or diversity were greater than the estimates in the SBE.

We additionally tested for the occurrence of Eurasian ancestry not present in the KhoeSan by considering Eurasian-KhoeSan source pairs in the tests. The Baphuthi, amaZulu, Zambian, and Zimbabweans did not produce results to support such admixture. The Duma San, amaNdebele, and Basotho recovered a common significant f_3 value, and in all cases the top results included GIH (Gujarati) or eastern Asians. The results here were not consistent across the Nguni speakers. All the southern KhoeSan produced significant results for this test and the top scores included a European source.

To provide chronological context to admixture events supported by the f_3 results, we estimated admixture dates with LD decay curves (MALDER, Table S5). We focus on events younger than 5 kya as beyond this the accuracy of the LD decay curve is questionable. Multiple admixture events were supported for the Baphuthi, amaZulu, Nama, Himba, and Zambians. In the Baphuthi we detected recent admixture between a KhoeSan and European group dating to ~1786 CE. Such dates may correspond to colonial era European admixture, as they are shared with events detected in the Karretjie, ‡Khomani, and Nama (1808–1835 CE), which are known to reflect recent admixture events.[3,17,63–65] Several of the southern SBE produced KhoeSan-European admixture dates

distinctly older (1450–908 CE), indicating an event not tied to the 17th century expansion of Europeans into southern Africa. We looked at date estimates in SBE groups not included in the focal populations to add context to these older events. The other SBE produced similar dates (Table S5), the oldest of which were similar to events detected in the Nama (252 CE–139 BCE). This suggests that the oldest Eurasian admixtures (~908 BCE) in the SBE may reflect Eurasian ancestry brought southward by events related to the arrival of the East African pastoralist groups.

Admixture between African populations were detected in the Baphuthi (991–812 CE) and in the amaZulu (1258–1143 CE), while older dates were detected for the more northerly Himba and Zambians (843 CE–118 BCE), suggesting more recent admixture for the groups further South.

To profile the extent of historic and/or ancient bottlenecks and inbreeding on the Baphuthi genome, we estimated the cRoH for five size categories.

The Baphuthi were notably different from the other southern SBE in the size categories reflective of recent inbreeding (RoH > 1 Mb; reflecting <10 generations ago, since ~1670 CE; see McQuillan et al.)[66] (Figure S7) and were more similar to the KhoeSan groups. Size categories reflective of ancient events (<1.5 Mb) were again more similar to the southern KhoeSan.

Overall results suggest that historic events, but not recent inbreeding/bottlenecks, were shared between the Baphuthi and SBE. Among the southern Bantu speakers, we note that the amaZulu and Himba showed high cRoH for the category reflective of ancient events. The Duma San and Himba had support for recent events too. However, it is unclear how different admixture histories have impacted these cRoH metrics.

Sex biases in admixture based on the X chromosome

We used the X chromosome ADMIXTURE components to detect possible sex biases in admixture by comparing them to the autosome as a ratio. We used the ratio of non-KhoeSan African:KhoeSan ancestry (NKS:KS) discussed below as $(NKS:KS)_{autosomal}:(NKS:KS)_{X\ chromosome}$.

At K = 5, major regional ancestries are represented and could be related easily to that observed in the autosome (Figure S8). Specifically, the autosome and X chromosome shared a non-KhoeSan African component (YRI), a KhoeSan component (Naro), an East Eurasian component (CDX), and a West Eurasian component (GBR or IBS).

Congruence between the datasets was supported by strong correlations of a single autosome-X chromosome pair (coefficient > 0.92, p value < 0.001, Figure S9).

The fifth components were not strongly correlated with each other nor with any other component. The fifth autosomal component, a possible eastern African component (Somali), was best correlated with the X chromosome's Naro and YRI components (coefficient < 0.15, p value < 0.01, Figure S9). The X chromosome's fifth component, a possible South Asian component (GIH), was weakly correlated with the autosomal GBR and CDX components (coefficient < 0.26, p value < 0.001, Figure S9). As these components were ambiguously related to other components, we discuss the less ambiguous YRI:Naro ratio for both autosomal and X chromosome results.

Now that we have confirmed that the autosomal and X chromosome components are comparable, we discuss the change in the non-KhoeSan African:KhoeSan ancestry ratios between autosome and X chromosome.

Firstly, on the basis of the ratio of the sum of the NKS and KS components, i.e., $(NKS+KS)_{autosomal}:(NKS+KS)_{X\ chromosome}$, we saw very little change in the amount of YRI+Naro ancestry across populations (Table S6). The exceptions were two eastern African groups (MKK and LWK) and the KhoeKhoe-speaking Hai||om where there was a decreased ratio, indicating a possible male bias on non-African admixture (Table S6). In these three populations, it is possible that non-African admixture may confound signals of sex-biased admixing.

The $(NKS:KS)_{autosomal}:(NKS:KS)_{X\ chromosome}$ ratios showed variation across Africa but were almost entirely above 1 (Table S6, Figure 4). Many populations were not statistically different from a ratio of 1, but this may be because of low power. Values larger than 1 indicate a female sex bias in KhoeSan admixture, which is wide-spread across communities. Of the southern African SBE, the Baphuthi had the largest ratio, on par with Bantu speakers further north (~1.76, p < 0.01), suggesting a stronger female bias in KhoeSan assimilation compared to the other two southern SBE (amaNdebele and Duma San, ratio ~1.25, not statistically different from 1). The Hai||om and Chrissie San both had values supporting a female KhoeSan bias (>1.19), suggesting that the bias was not restricted to the Bantu-speaking communities.

Discussion

The Baphuthi have an oral history of descent from BaTwa/Baroa (KhoeSan) and a more recent narrative of the assimilation of refugees.[35,43] The results from our investigation show that the Baphuthi have an overall similarity to neighboring Bantu-speaking communities but are indeed unique in some aspects of their genetic history. We found no support for a unique eastern KhoeSan ancestry, but the Baphuthi appear to have a unique drifted genome.

The Baphuthi have close affinities to the southern Bantu-speaking communities

The Baphuthi show strong genetic affinities to the communities from the surrounding region who speak southern Bantoid, eastern Bantu (SBE) languages as we may expect from their history. This was evident from the overlapping positions in the PCA, similar ratio of ADMIXTURE components related to southern Bantu-speakers and KhoeSan (~5:1) as previously reported,[47,67] and from the MALDER and f_3 results. The profile was particularly similar to the

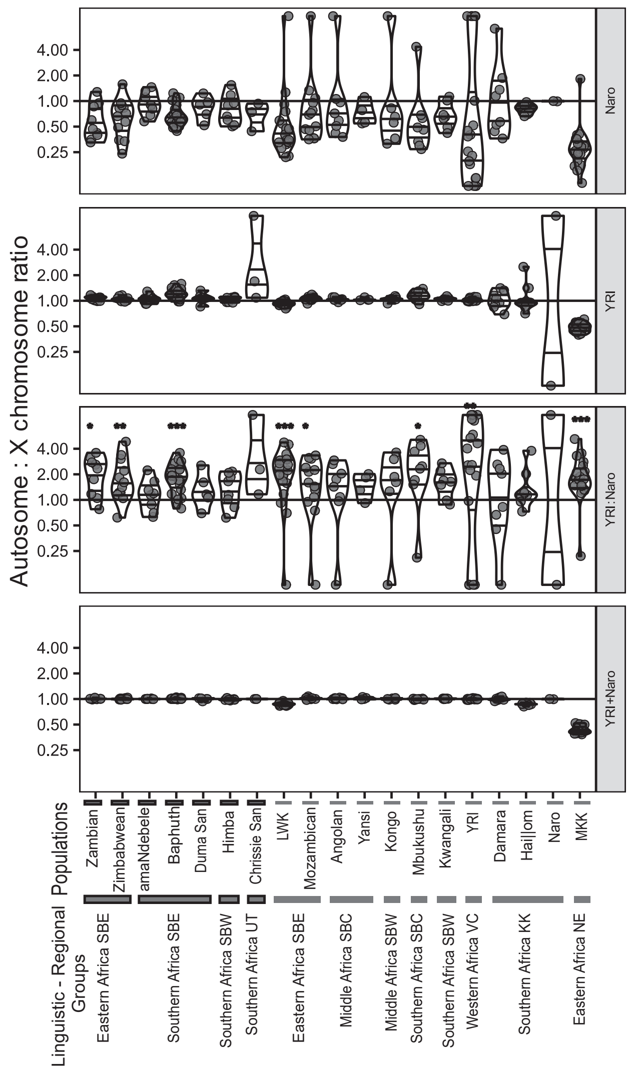

Figure 4. Ratio of autosomal:X chromosome ADMIXTURE components for African populations

Components shown are the KhoeSan (Naro) and non-KhoeSan African (YRI), the ratio of the two (YRI:Naro), and the sum (YRI + Naro) at K = 5. Horizontal lines in violin plots indicate the 25%, 50%, and 75% quantiles. Note that the y axis is on a binary log scale and all values were capped at y = 10 to aid visualization. Horizontal line at y = 1 indicates the expected value when the autosome and X chromosome are equal. Asterisk on YRI:Naro subplot indicates distributions statistically different from y = 1; *$p < 0.05$, **$p < 0.01$, ***$p < 0.001$. Highlighted bars below the plot indicate the focal populations.

amaZulu in our analysis and in previous work.[8,23,45] In contrast, Baphuthi notably differed from the Duma San (Nguni speakers) even though both share a narrative of recent KhoeSan descent.[7,8]

The Baphuthi genetic history appears to have been shaped by bottlenecks and/or inbreeding not shared with other SBE communities as shown by the cRoH profiles. However, these events are not recent.

While the Baphuthi speak Siphuthi, a Southern/Lowland Ndebele language[41] (web resources), we found the amaNdebele shared more characteristics with the Basotho than the Baphuthi. This was despite the fact that the Baphuthi and amaZulu were geographically and linguistically closer to amaNdebele (and Duma San). Clearly the relationship among the Nguni-speaking communities is not simple.

The Nguni-speakers are suspected to have been the earliest of the present-day Ntu-speaking agriculturalists to arrive to southern Africa[68] and their relatedness may give details to the early history of the region. The divergence of the amaNdebele from the other Nguni may result from non-uniform admixture between the Nguni and later arriving communities. The cultural differences of the Nguni and Sotho-Tswana provide some clues as the customs unique to the Nguni in southern Africa have possible parallels in Rwanda. These include "hlonipha" (respectful etiquette) and a distinct dichotomy in social roles (e.g., between men tending to cattle while women tend to crops),[40] suggesting the arrival of an "intervening influence" (possibly related to the Basotho) between the regions.

The amaNdebele and Duma San showed support for additional Eurasian and KhoeSan admixture events not seen in the amaZulu and Baphuthi (Tables S2–S4) but in common with the Basotho.

While the amaNdebele migrated out of KwaZulu-Natal during the 1600s[40] and may have admixed during this migration, the east African/Eurasian ADMIXTURE components are too consistent across individuals to be from recent admixture and there was no support from f_3 and MALDER (Tables 1 and S4). Instead the contribution may derive from admixture with another SBE group. The same may be said for the Duma San.

The Basotho appear to descend from recent admixture between a southern SBE with a group from further north. Indeed the Basotho could be modeled as f_3 (Baphuthi, Zambians; Basotho) or f_3 (Duma San, Zimbabweans; Basotho) (but no other combination of these, Table S4). This result would relate to the elevated western Bantu contribution in the Zambians and Duma San (increased "Himba" and "Naro" ADMIXTURE components, Table S3). The detected admixture dates to 1450–1173 CE (Table 1), which would reflect a late Iron-Age contact. Our results support that the "proto-Basotho" migrated southward as one would expect as Sesotho is related to Sepedi, Setswana, Tshivenda, and Makua, found predominantly further north (web resources).[69]

The origin of the SBW component in the Basotho (possibly related to that in the Zambians) may be tied to the arrival of Benfica pottery tradition in southern Congo and ultimately a possible western Bantu source.[70,71] Our MALDER dates for such an event, 843 CE–118 BCE, coincide with the establishment of early Iron-Age Benfica pottery in northern Botswana (150–350 CE).[72] This may be the source of the components absent in the Baphuthi.

A complex "KhoeSan" descent

In common with other Bantu-speaking communities, the Baphuthi retain oral history of KhoeSan descent and

Table 1. Admixture event dates estimated across populations on the basis of LD decay curves

		Amplitude					Date (generations ago)				Date (CE)		
**Linguistic group**	**Population**	μ	**SD**	***Z* score**	**Source 1**	**Source 2**	μ	**SD**	**Lower bound**	**Upper bound**	μ	**Lower bound**	**Upper bound**
Taa	Karretjie	1.18E−03	3.99E−05	29.55	Ju/'hoansi	GBR	4.88	0.34	4.53	5.22	1,823	1,833	1,814
	‡Khomani	1.19E−03	6.07E−05	19.62	Ju/'hoansi	GBR	5.04	0.52	4.52	5.56	1,819	1,833	1,804
Khoe-Kwadi	Nama	8.38E−04	5.46E−05	15.34	Ju/'hoansi	TSI	67.98	6.99	60.99	74.97	57	252	−139
		5.65E−04	3.84E−05	14.71	Ju/'hoansi	GBR	4.95	0.47	4.48	5.42	1,821	1,835	1,808
SBE - Nguni	Baphuthi	3.33E−04	6.83E−05	4.88	Ju/'hoansi	TSI	401.19	99.14	302.05	500.33	−9,273	−6,497	−12,049
		3.19E−05	8.72E−06	3.70	Ju/'hoansi	TSI	6.20	1.69	4.51	7.89	1,786	1,834	1,739
		2.59E−04	1.19E−05	21.84	Ju/'hoansi	YRI	37.80	3.21	34.59	41.00	902	991	812
	amaNdebele	3.36E−04	1.94E−05	17.28	Ju/'hoansi	IBS	34.10	3.46	30.64	37.56	1,005	1,102	908
	amaZulu	3.68E−04	3.81E−05	9.70	Ju/'hoansi	IBS	299.05	41.15	257.90	340.20	−6,413	−5,261	−7,566
		2.18E−04	1.26E−05	17.33	Ju/'hoansi	Mozambique	27.12	2.06	25.05	29.18	1,201	1,258	1,143
SBE - Non-Nguni	DumaSan	2.99E−04	3.56E−05	8.39	Ju/'hoansi	IBS	23.12	4.91	18.21	28.03	1,313	1,450	1,175
	Basotho	2.89E−04	1.48E−05	19.57	Ju/'hoansi	GBR	25.57	2.55	23.02	28.13	1,244	1,315	1,172
	Zambian	3.77E−04	3.41E−05	11.04	Ju/'hoansi	CDX	383.28	47.15	336.13	430.44	−8,772	−7,452	−10,092
		6.19E−05	1.63E−05	3.80	Ju/'hoansi	Himba	57.06	17.16	39.90	74.21	362	843	−118
	Zimbabwean	2.51E−04	2.29E−05	10.97	Yansi	TSI	268.26	24.35	243.91	292.61	−5,551	−4,869	−6,233
SBW	Himba	4.72E−04	1.55E−04	3.04	Xun	GBR	540.40	155.02	385.38	695.41	−13,171	−8,831	−17,512
		1.45E−04	1.29E−05	11.22	Ju/'hoansi	Damara	49.19	6.06	43.14	55.25	583	752	413

Abbreviations: μ, mean; SD, standard deviation; CE, Common Era. Linguistic abbreviations used: southern Bantoid, western Bantu (SBW); southern Bantoid, eastern Bantu (SBE).

culture, which is reflective of KhoeSan influence.[7,8,73] We found support for a common genetic origin of the KhoeSan heritage with that of the southern SBE and southern KhoeSan but no support for a unique KhoeSan descent in the Baphuthi. In some SBE (e.g., Zambians), KhoeSan descent was detected that was distinct from the southern SBE. The Baphuthi were not among these groups, contrary to what was anticipated from the "essentialist" reading of "Bushman" descent in historic texts. Moreover, the component prevalent in the Baphuthi was most prevalent in the Karretjie, ‡Khomani, and Chrissie San suggesting a regional affinity. Our discussion is made with the caveat that a higher resolution dataset could possibly detect signals that are elusive in the current study.

The KhoeSan ADMIXTURE component, referred to as the "Naro" component, is seen in the Baphuthi and the southern SBE, Duma San, and Lake Chrissie San. The southern SBE lacked a clear difference from the southern KhoeSan based on the f_3 statistics and among the southern SBE, the "Baphuthi":"Naro" ADMIXTURE ratios were remarkably consistent. This is particularly noteworthy considering the variation in linguistic affinities and other ancestral components of the southern SBE. These results support the proposed common source and event for the KhoeSan admixture in the region.[9,74] The X chromosome deviated from the proportions detected in the autosome and supported a by and large female bias in KhoeSan admixture (Figure 4), in line with earlier work.[22,71,74,72] The trend may be related to the socio-political dynamics during the Bantu-language expansion.

When compared to other SBE, the southern SBE have elevated KhoeSan components and a weaker bias for female KhoeSan admixture, a pattern shared with groups from south-west Africa (Figure 4). This is often argued as the result of demographic dynamics when the early Iron Age Bantu-expansion progressed across in southern Africa.[74,75,76] The change in environmental conditions would have slowed the rate of population growth and allowed for greater admixture with local populations,[77] leading to greater KhoeSan ancestry and potentially more equal assimilation of sexes.

If we assume that the KhoeSan affinities of the Baphuthi indeed reflect a recently acquired ancestry from the Maloti-Drakensberg, these ancestors would then have had shared affinities with other groups across the region. The broad relatedness across the region is possible as the ancestral languages of the groups in which the "Naro" ADMIXTURE component was largest (G|ui and G||ana, the Karretjie [likely |Xam language], the ||Xegwi [Chrissie San ancestors]) belonged to the !Ui branch of the Tuu family.[8] The languages spoken around the Drakensberg are likely to have also been Tuu.[78]

The southern SBE and southern KhoeSan appear to have a common ancestral KhoeSan population, but the admixture history is not likely shared. The variation of the "Baphuthi":"Naro" ADMIXTURE ratios among the southern KhoeSan suggests independent admixture events, contrasting to the common ancestral "proto-southern SBE" who drifted or admixed to give rise to the present-day SBE diversity.

Some traits make the Baphuthi data peculiar from other SBE. Of particular interest is that the Bantu-related "Baphuthi" and "Naro" ADMIXTURE components are at higher proportions compared to other southern SBE, yet several Bantu-related components are frequently absent from the Baphuthi (Figure S5). The "Baphuthi" and "Naro" components sum to >95% for most Baphuthi but none of the other southern SBE (median total of the two components ~85%–89%, Figure S5). Furthermore, components such as the "Himba," "Kalenjin," and "Jola," which were almost ubiquitous in the southern SBE, were frequently absent from the Baphuthi. The Baphuthi have apparently diverged even from their closest genetic kin, the amaZulu. For perspective, consider that only the Lake Chrissie San, ‡Khomani, Karretjie, and G|ui and G||ana had individuals with similar profiles (i.e., the sum of the two components >90%). This may be related to the historic and ancient bottlenecks detected in the Baphuthi (cRoH and PCA results). This distinction is surprising, as the Baphuthi's history mentions the historic amalgamation of diverse SBE groups; e.g., amaZizi, Maphuthing, Bafokeng, and Mpondomise.[43] Furthermore, the Baphuthi had evidence for KhoeSan sex bias more similar to eastern Africans than their geographic neighbors (Figure 4). There is no support for a recent connection of the Baphuthi to east Africa based on the autosome. It also seems unlikely that the sex bias is a retained ancestral signal as this would contradict the overall regional pattern and indeed a pattern shared with the Chrissie San and Hai||om. Most likely there has been some distortion that may be due to drift and the lower N_e of the X chromosome. Without more Nguni populations, our discussion of the Baphuthi's admixture biases is necessarily inconclusive.

With regards to the minor autosomal components absent in the Baphuthi, an independent loss of the same in the Baphuthi, southern KhoeSan, and G|ui and G||ana seems unlikely considering the large differences in the proportions of the two major components if the minor components were at notable levels in the source populations. The position of the Baphuthi on PC3 (Figure S1) and the high cRoH supports a bottleneck in the Baphuthi related to southern Bantu-speaking and KhoeSan affinities. Moreover, the cRoH estimates did not support a recent shared bottleneck for the Baphuthi and amaZulu, indicating that the lower proportions in the amaZulu are not the result of the recent bottleneck seen in the Baphuthi. It may be the case that these minor components had already drifted, or were not present, in the early arriving Bantu-speaking communities.

A separate (re)introduction of the minor components can be supported. The amaZulu and Baphuthi could not be modeled as an admixture of east Africans and a KhoeSan group, and f_3 suggested that the east African ancestry detected in the Basotho, Duma San, and

amaNdebele (e.g., the Asian admixture on f_3 and the "Somali," "Kalenjin," "Himba" ADMIXTURE components, Figure 2, Table S4) may reflect subsequent admixture rather than recent drift in the Nguni. The Duma San have a long history with the amaZulu but also acknowledge more recent Bantu-speaker ancestors[8] that may be the source of the (re)introduced components.

The possible re-introduction parallels the argument for multiple streams of east African migration into southern Africa (i.e., Chifumbaze complex)[70,71] with an early arriving Iron Age Bantu-related community, possibly Kwale tradition facies (here the "proto-southern SBE" ADMIXTURE profile of the Baphuthi or amaZulu) and then a replacement or assimilation by late Iron Age groups possibly derived from early Iron Age Kalundu tradition ceramic facies or possibly Nkope influence (marked by the addition of the "Himba," "Kalenjin," and "Jola" ADMIXTURE components as in Banyarwanda, Barundi in Figure 2). MALDER date estimates for admixture between KhoeSan and non-KhoeSan Africans (991 CE–118 BCE in the Zambians, Himba, and Baphuthi) pre-date the arrival of the late Iron Age Bantu expansion into south-eastern Africa,[21,24] while dates detected in the other southern SBE (1102–908 CE amaNdebele; 1450–1143 CE amaZulu, Duma San, Basotho) for admixture between KhoeSan and non-KhoeSan could be related to the second millennium CE late Iron Age expansion.[79]

We found no "ancient" distinction of the Baphuthi from the SBE in KhoeSan ancestry, but the Baphuthi may have recently incorporated amaTola.[42,43] The amaTola reportedly incorporated Khoekhoe during the Frontier wars (1779–1878 CE) as well as Bantu-speakers[7] and in the amaTola descendants, the Duma San,[8] we do see greater proportions of ADMIXTURE components in common with the Nama (a Khoekhoe group). While Khoekhoe pastoralists are recorded along the western parts of South Africa[14] and left cognates in the Nguni languages,[68] the extent of their range eastward is unclear.[80] The Nama have genetic ancestry indicating an admixture between a southern KhoeSan group and a Eurasian group related to the arrival of pastoralism in the region[9,65] possibly 252 CE–139 BCE (Table 1).

If we assume that the east African components identified in the southern SBE entered during their migration through east Africa into southern Africa,[79–81] then the absence of elevated east African ancestry in the Baphuthi would indicate that the assimilated "Khoekhoe" were perhaps culturally pastoralists but not of Khoe-Kwadi descent. Alternatively, that component might have been lost to genetic drift. Further work will help clarify this issue.

In this work, we investigated to what extent the oral history of Baphuthi as eastern San and Khoekhoe descendants is reflected in their genetics. The Baphuthi harbor signals for an interesting connection to the early arrival of the Bantu languages, but we could not support a unique eastern KhoeSan contribution. In the case of the Baphuthi and the previously investigated Duma San, we find some support for KhoeSan descent but not the "essentialist" reading of an eastern KhoeSan from historic texts. Such essentialist interpretations have created misconstrued narratives of ethnic/biological distinctions. The high status attained by KhoeSan Shaman and the pride taken in the "Bushman" means of semi-nomadic subsistence[7] may have entrenched the importance of "Bushman" heritage in the collective memory and perhaps without necessarily reflecting recent assimilation of some remnant KhoeSan group.

Data and code availability

Ethics approval and participant consent allows for the data presented in this study to be used in future research provided the research abides by the agreement in the original consent forms and ethics application. To avoid any conflicts of interest and violations of the ethics agreement, the request for data sharing will be conditioned upon signing a Data Transfer Agreement. Further details are provided in Note S2. Array data has been deposited at the European Genome-phenome Archive (EGA), which is hosted by the EBI and the CRG, under accession numbers EGA: EGAD00010002467 and EGAD00010002468. Further information about EGA can be found on https://ega-archive.org.

Supplemental information

Supplemental information can be found online at https://doi.org/10.1016/j.ajhg.2023.03.018.

Acknowledgments

We thank all the participants for their time and engagement. R.J.D. thanks the South African National Research Foundation, the Commonwealth Commission in the U.K., Santander Travel grant and the Oppenheimer Memorial Trust for support. The project was funded by a Commonwealth Commission in the U.K. and Boise Trust Fund award to R.J.D. The funders had no role in study design, data collection, analyses, decision to publish, preparation of the manuscript, or the outcome. We thank Alessandro Raveane for useful discussions and assistance. All data analyses were performed at the ILIFU High-Performance Computer Centre of the University of the Western Cape, South Africa (https://www.ilifu.ac.za/). This study makes use of data generated or collated by the African Partnership for Chronic Disease Research. A full list of the investigators and funders who contributed to the generation or collation of the African Partnership for Chronic Disease Research data is available from www.apcdr.org.

Declaration of interests

The authors declare no competing interests.

Received: May 25, 2022
Accepted: March 27, 2023
Published: April 26, 2023

Web resources

African Genome Variation Project, https://ega-archive.org/dacs/EGAC00001000237

Census 2011, Statistics South Africa, http://www.statssa.gov.za/census/census_2011/census_products/Census_2011_Census_in_brief.pdf

Glottolog 4.0., https://glottolog.org/

Human Evolutionary Genetics Group Data, https://capelligroup.wordpress.com/data

Pickrell Data, https://reich.hms.harvard.edu/datasets

PMCMRplus, https://CRAN.R-project.org/package=PMCMRplus

R: A Language and Environment for Statistical Computing, https://www.r-project.org/

R package "corrplot," https://github.com/taiyun/corrplot

Schlebush Data, http://jakobssonlab.iob.uu.se/data

The 1000 Genomes Project, http://www.internationalgenome.org/home

References

1. Kent, S. (2002). Interethnic encounters of the first kind: an introduction. In Ethnicity, Hunter-Gatherers, and the "Other": Association or Assimilation in Africa, S. Kent, ed. (Smithsonian Institution Press).
2. Race Ethnicity and Genetics Working Group, Bonham, V., Boyer, J., Brody, L., Brooks, L., Collins, F., Guttmacher, A., McEwen, J., Muenke, M., Olson, S., et al. (2005). The Use of Racial, Ethnic, and Ancestral Categories in Human Genetics Research. Am. J. Hum. Genet. *77*, 519–532.
3. Morris, A.G. (2008). Searching for 'real' Hottentots: the Khoekhoe in the history of South African physical anthropology. S. Afr Humanit *20*, 221–233.
4. Barbujani, G., and Colonna, V. (2010). Human genome diversity: frequently asked questions. Trends Genet. *26*, 285–295.
5. Chennells, R., and Steenkamp, A. (2016). International Genomics Research involving the San People. In Ethics Dumping" – Paradigmatic Case Studies: A report for TRUST, D. Schroeder, J.C. Lucas, S. Fenet, and F. Hirsch, eds. (TRUST Project), pp. 35–40.
6. Schlebusch, C. (2010). Issues raised by use of ethnic-group names in genome study. Nature *464*, 487.
7. Challis, S. (2016). Re-tribe and resist : the ethnogenesis of a creolised raiding band in response to colonisation. In Tribing and Untribing the Archive: Identity and the material record in southern KwaZulu-Natal in the Late Independent and Colonial periods, C. Hamilton and N. Leibhammer, eds. (University Kwazulu Natal Press), pp. 282–299.
8. Schlebusch, C.M., Prins, F., Lombard, M., Jakobsson, M., and Soodyall, H. (2016). The disappearing San of southeastern Africa and their genetic affinities. Hum. Genet. *135*, 1365–1373.
9. Montinaro, F., Busby, G.B.J., González-Santos, M., Oosthuitzen, O., Oosthuitzen, E., Anagnostou, P., Destro-Bisol, G., Pascali, V.L., and Capelli, C. (2017). Complex ancient genetic structure and cultural transitions in southern African populations. Genetics *205*, 303–316.
10. Halford, S.J. (1949). The Griquas of Griqualand (Juta and Company, Ltd.).
11. King, R. (2017). Living on edge: new perspectives on anxiety, refuge and colonialism in Southern Africa. Camb. Archaeol. J. *27*, 533–551.
12. How, M.W. (1962). The Mountain Bushmen of Basutoland (J. L. Van Schaik).
13. Vinnicombe, P. (1976). People of the Eland: Rock Paintings of the Drakensberg Bushmen as a Reflection of Their Life and Thought (University of Natal Press).
14. Klinghardt, G.P., and Barnard, A. (1993). Hunters and HErders of Southern Africa. A comparative ethnography of the Khoisan Peoples. S. Afr. Archaeol. Bull. *48*, 54.
15. Adhikari, M. (2005). Not White Enough, Not Black Enough: Racial Identity in the South African Coloured Community (Double Storey Books and Athens).
16. Challis, S. (2018). Creolization in the investigation of rock art of the colonial era. In The Oxford Handbook of the Archaeology and Anthropology of Rock Art, B. David and I. McNiven, eds. (Oxford University Press), pp. 611–633.
17. Schlebusch, C.M., Skoglund, P., Sjödin, P., Gattepaille, L.M., Hernandez, D., Jay, F., Li, S., De Jongh, M., Singleton, A., Blum, M.G.B., et al. (2012). Genomic variation in Seven Khoe-san groups reveals adaptation and complex african history. Science *338*, 374–379.
18. Prins, F.E. (2009). Secret San of the Drakensberg and their rock art legacy. Crit. Arts *23*, 190–208.
19. Pickrell, J.K., Patterson, N., Barbieri, C., Berthold, F., Gerlach, L., Güldemann, T., Kure, B., Mpoloka, S.W., Nakagawa, H., Naumann, C., et al. (2012). The genetic prehistory of southern Africa. Nat. Commun. *3*, 1143.
20. Montinaro, F., and Capelli, C. (2018). The evolutionary history of Southern Africa. Curr. Opin. Genet. Dev. *53*, 157–164.
21. Oliver, R. (1966). The Problem of the Bantu Expansion. J. Afr. Hist. *7*, 361–376.
22. de Filippo, C., Barbieri, C., Whitten, M., Mpoloka, S.W., Gunnarsdóttir, E.D., Bostoen, K., Nyambe, T., Beyer, K., Schreiber, H., de Knijff, P., et al. (2011). Y-Chromosomal Variation in Sub-Saharan Africa: Insights Into the History of Niger-Congo Groups. Mol. Biol. Evol. *28*, 1255–1269.
23. Busby, G.B., Band, G., Si Le, Q., Jallow, M., Bougama, E., Mangano, V.D., Amenga-Etego, L.N., Enimil, A., Apinjoh, T., Ndila, C.M., et al. (2016). Admixture into and within sub-Saharan Africa. Elife *5*, 1–44.
24. Skoglund, P., Thompson, J.C., Prendergast, M.E., Pinhasi, R., Krause, J., Reich, D., Mittnik, A., Morris, A.G., and Boivin, N. (2017). Reconstructing Prehistoric African Population Structure. Cell *171*, 59–71.
25. Sinclair-Thomson, B., and Challis, S. (2020). Runaway slaves, rock art and resistance in the Cape Colony, South Africa. Azania *55*, 475–491.
26. Skotnes, P. (2007). Claim to the Country: The Archive of Lucy Lloyd and Wilhelm Bleek (Jacana Media).
27. Mitchell, P. (2005). Modeling Later Stone Age Societies in Southern Africa. In African archaeology: a critical Introduction, A.B. Stahl, ed. (Wiley-Blackwell), pp. 150–173.
28. Heese, H. (1984). Groep sonder grense: Die rol en status van die gemengde bevolking aan die Kaap (Protea Boekhuis), pp. 1652–1795.
29. Ross, R. (1984). Cape of Torments: Slavery and Resistance in South Africa (Routledge & Kegan Paul Press).
30. Schuster, S.C., Miller, W., Ratan, A., Tomsho, L.P., Giardine, B., Kasson, L.R., Harris, R.S., Petersen, D.C., Zhao, F., Qi, J., et al. (2010). Complete Khoisan and Bantu genomes from southern Africa. Nature *463*, 943–947.
31. Wells, L.H. (1960). Bushman and Hottentot statures: A review of the evidence. S Afr J Sci *56*, 277–281.

32. Goldsby, R.A. (1971). Race and Races (Macmillan Publishing Co.).
33. Coon, C.S. (1973). The Living Races of Man (Random House).
34. Wright, J.B. (1971). Bushman Raiders of the Drakensberg (University of Natal Press), pp. 1840–1870.
35. King, R., and Challis, S. (2017). The 'interior world' of the nineteenth-century Maloti-Drakensberg mountains. J. Afr. Hist. *58*, 213–237.
36. Challis, S. (2012). Creolisation on the nineteenth-century frontiers of Southern Africa: A case study of the AmaTola 'Bushmen' in the Maloti-Drakensberg. J. South Afr. Stud. *38*, 265–280.
37. Wright, J.B. (2007). Bushman raiders revisited. In Claim to the country: the archive of Lucy Lloyd and Wilhelm Bleek (Jacana Media), pp. 119–129.
38. Traill, A. (1995). Language and Social History, Cape Town. In The Khoesan languages of South Africa, R. Mesthrie, ed. (David Philip), pp. 1–18.
39. Ellenberger, D.F. (1969). History of the Basuto, Ancient and Modern: Third Period (Negro Universities Press).
40. Huffman, T.N. (2004). The archaeology of the Nguni past. S. Afr Humanit *16*, 79–111.
41. Donnelly, S. (1999). Southern Tekela Nguni is alive: reintroducing the Phuthi language. Int. J. Soc. Lang. *136*, 97–120.
42. Victor, E. (1953). La fin tragique des bushmen - les derniers hommes vivants de l'age de la pierre (Amiot-Dumont).
43. King, R. (2014). The BaPhuthi Chiefdom, Cattle Raiding, and Colonial Rule in Nineteenth-Century Southern Africa. PhD Thesis (Oxford University).
44. Jolly, P. (1996). Symbiotic Interaction Between Black Farmers and South-Eastern San: Implications for Southern African Rock Art Studies, Ethnographic Analogy, and Hunter-Gatherer Cultural Identity. Curr. Anthropol. *37*, 277–305.
45. Gurdasani, D., Carstensen, T., Tekola-Ayele, F., Pagani, L., Tachmazidou, I., Hatzikotoulas, K., Karthikeyan, S., Iles, L., Pollard, M.O., Choudhury, A., et al. (2015). The African Genome Variation Project shapes medical genetics in Africa. Nature *517*, 327–332.
46. 1000 Genomes Project Consortium, Abecasis, G.R., Auton, A., Brooks, L.D., DePristo, M.A., Durbin, R.M., Handsaker, R.E., Kang, H.M., Marth, G.T., McVean, G.A., et al. (2012). An integrated map of genetic variation from 1,092 human genomes. Nature *491*, 56–65.
47. González-Santos, M., Montinaro, F., Oosthuizen, O., Oosthuizen, E., Busby, G.B.J., Anagnostou, P., Destro-Bisol, G., Pascali, V., and Capelli, C. (2015). Genome-Wide SNP Analysis of Southern African Populations Provides New Insights into the Dispersal of Bantu-Speaking Groups. Genome Biol. Evol. *7*, 2560–2568.
48. Purcell, S., Neale, B., Todd-Brown, K., Thomas, L., Ferreira, M.A.R., Bender, D., Maller, J., Sklar, P., de Bakker, P.I.W., Daly, M.J., and Sham, P.C. (2007). PLINK: A Tool Set for Whole-Genome Association and Population-Based Linkage Analyses. Am. J. Hum. Genet. *81*, 559–575.
49. Patterson, N., Price, A.L., and Reich, D. (2006). Population structure and eigenanalysis. PLoS Genet. *2*, e190–e2093.
50. Novembre, J., Johnson, T., Bryc, K., Kutalik, Z., Boyko, A.R., Auton, A., Indap, A., King, K.S., Bergmann, S., Nelson, M.R., et al. (2008). Genes mirror geography within Europe. Nature *456*, 98–101.
51. Alexander, D.H., Novembre, J., and Lange, K. (2009). Fast Model-Based Estimation of Ancestry in Unrelated Individuals. Genome Res. *19*, 1655–1664.
52. Pritchard, J.K., Stephens, M., and Donnelly, P. (2000). Inference of population structure using multilocus genotype data. Genetics *155*, 945–959.
53. Kopelman, N.M., Mayzel, J., Jakobsson, M., Rosenberg, N.A., and Mayrose, I. (2015). Clumpak: a program for identifying clustering modes and packaging population structure inferences across K. Mol. Ecol. Resour. *15*, 1179–1191.
54. Wickham, H. (2009). ggplot2: Elegant Graphics for Data Analysis (Springer-Verlag).
55. Patterson, N., Petersen, D.C., van der Ross, R.E., Sudoyo, H., Glashoff, R.H., Marzuki, S., Reich, D., and Hayes, V.M. (2010). Genetic structure of a unique admixed population: implications for medical research. Hum. Mol. Genet. *19*, 411–419.
56. Peter, B.M. (2016). Admixture, population structure, and f-statistics. Genetics *202*, 1485–1501.
57. Patterson, N., Moorjani, P., Luo, Y., Mallick, S., Rohland, N., Zhan, Y., Genschoreck, T., Webster, T., and Reich, D. (2012). Ancient admixture in human history. Genetics *192*, 1065–1093.
58. Loh, P.R., Lipson, M., Patterson, N., Moorjani, P., Pickrell, J.K., Reich, D., and Berger, B. (2013). Inferring admixture histories of human populations using linkage disequilibrium. Genetics *193*, 1233–1254.
59. Brucato, N., Kusuma, P., Beaujard, P., Sudoyo, H., Cox, M.P., and Ricaut, F.X. (2017). Genomic admixture tracks pulses of economic activity over 2,000 years in the Indian Ocean trading network. Sci. Rep. *7*, 2919–3010.
60. Ongaro, L., Molinaro, L., Flores, R., Marnetto, D., Capodiferro, M.R., Alarcón-Riquelme, M.E., Moreno-Estrada, A., Mabunda, N., Ventura, M., Tambets, K., et al. (2021). Evaluating the Impact of Sex-Biased Genetic Admixture in the Americas through the Analysis of Haplotype Data. Genes *12*, 1580.
61. Rosenberg, N.A., Pritchard, J.K., Weber, J.L., Cann, H.M., Kidd, K.K., Zhivotovsky, L.A., and Feldman, M.W. (2002). Genetic Structure of Human Populations. Science *298*, 2381–2385.
62. Bryc, K., Auton, A., Nelson, M.R., Oksenberg, J.R., Hauser, S.L., Williams, S., Froment, A., Bodo, J.-M., Wambebe, C., Tishkoff, S.A., and Bustamante, C.D. (2010). Genome-wide patterns of population structure and admixture in West Africans and African Americans. Proc. Natl. Acad. Sci. USA *107*, 786–791.
63. Güldemann, T. (2008). A linguist's view: Khoe-Kwadi speakers as the earliest food-producers of southern Africa. South. Afr. Humanit. *20*, 93–132.
64. Güldemann, T., and Stoneking, M. (2008). A historical appraisal of clicks: A linguistic and genetic population perspective. Annu. Rev. Anthropol. *37*, 93–109.
65. Pickrell, J.K., Patterson, N., Loh, P.-R., Lipson, M., Berger, B., Stoneking, M., Pakendorf, B., and Reich, D. (2014). Ancient west Eurasian ancestry in southern and eastern Africa. Proc. Natl. Acad. Sci. USA *111*, 2632–2637.
66. McQuillan, R., Leutenegger, A.L., Abdel-Rahman, R., Franklin, C.S., Pericic, M., Barac-Lauc, L., Smolej-Narancic, N., Janicijevic, B., Polasek, O., Tenesa, A., et al. (2008). Runs of Homozygosity in European Populations. Am. J. Hum. Genet. *83*, 359–372.
67. Choudhury, A., Ramsay, M., Hazelhurst, S., Aron, S., Bardien, S., Botha, G., Chimusa, E.R., Christoffels, A., Gamieldien, J., Sefid-Dashti, M.J., et al. (2017). Whole-genome sequencing for an enhanced understanding of genetic variation among South Africans. Nat. Commun. *8*, 2062.
68. Ownby, C.P. (1981). Early Nguni History: Linguistic Suggestions. S. Afr. J. Afr. Lang. *1*, 60–81.

69. Hiernaux, J. (2009). Bantu Expansion : The Evidence from Physical Anthropology Confronted with Linguistic and Archaeological Evidence. J. Afr. Hist. *9*, 505–515.
70. Huffman, T.N. (1989). Ceramics, settlements and Late Iron Age migrations. Afr. Archaeol. Rev. *7*, 155–182.
71. Huffman, T.N. (2021). Bambata pottery and Western and Bantu: re-interpreting the Early and Iron Age and in southern Africa. S. Afr Humanit *13*, 1–17.
72. Barbieri, C., Butthof, A., Bostoen, K., and Pakendorf, B. (2013). Genetic perspectives on the origin of clicks in Bantu languages from southwestern Zambia. Eur. J. Hum. Genet. *21*, 430–436.
73. Argyle, J. (1994). Khoisan-Southern Bantu Livestock Exchanges: Reinterpreting the linguistic evidence (summary). Azania *29-30*, 199.
74. Marks, S.J., Montinaro, F., Levy, H., Brisighelli, F., Ferri, G., Bertoncini, S., Batini, C., Busby, G.B.J., Arthur, C., Mitchell, P., et al. (2015). Static and Moving Frontiers: The Genetic Landscape of Southern African Bantu-Speaking Populations. Mol. Biol. Evol. *32*, 29–43.
75. Barham, L., and Mitchell, P. (2008). The First Africans: African Archaeology from the Earliest Toolmakers to Most Recent Foragers (Cambridge University Press).
76. Bostoen, K., Clist, B., Doumenge, C., Grollemund, R., Hombert, J.M., Muluwa, J.K., and Maley, J. (2015). Middle to Late Holocene Paleoclimatic Change and the Early Bantu Expansion in the Rain Forests of Western Central Africa. Curr. Anthropol. *56*, 354–384.
77. Quilodrán, C.S., Nussberger, B., Montoya-Burgos, J.I., and Currat, M. (2019). Hybridization and introgression during density-dependent range expansion: European wildcats as a case study. Evolution *73*, 750–761.
78. Mitchell, P. (2010). Genetics and southern African prehistory: An archaeological view. J. Anthropol. Sci. *88*, 73–92.
79. Holden, C.J. (2002). Bantu language trees reflect the spread of farming across sub-Saharan Africa: a maximum-parsimony analysis. Proc. Biol. Sci. *269*, 793–799.
80. Ehret, C. (1982). The first spread of food production to southern Africa. In The Archaeological and Linguistic Reconstruction of African History, C. Ehret and M. Posnansky, eds. (University of California Press), pp. 158–181.
81. Guthrie, M. (1971). Comparative Bantu: An Introduction to the Comparative Linguistics and Prehistory of the Bantu Languages (Gregg).

The American Journal of Human Genetics, Volume *110*

Supplemental information

Genetic heritage of the Baphuthi highlights an over-ethnicized notion of “Bushman” in the Maloti-Drakensberg, southern Africa

Ryan Joseph Daniels, Maria Eugenia D'Amato, Mpasi Lesaoana, Mohaimin Kasu, Karen Ehlers, Paballo Abel Chauke, Puseletso Lecheko, Sam Challis, Kirk Rockett, Francesco Montinaro, Miguel González-Santos, and Cristian Capelli

1 Supplemental Notes

Supplemental Note S1: Discussion on the use of population 'labels'

On the backdrop of colonial era racism, slavery, and genocide, people are rightfully sensitive about the words used to discuss them [1-3]. Participants in research should feel respected and that their sentiments are understood both to encourage ongoing public trust and for growth within the scientific community.

Terms used in our paper, such as ‘Hottentot’, ‘Khoisan’, ‘Bantu’ and ‘Bushman’ have a negative connotation to many who have been presently or historically referred to as such [4, 5]. All identifiers or ethnic labels carry some negative connotation because conflict with external communities is unavoidable. As such it is very challenging to find non-offensive terms when referring to communities and moreover when attempting to co-ordinate the use of terms across regions where histories are different. For example, while is South Africa the term ‘Bushman’ is strongly derogatory, some Kalahari hunter-gatherers prefer the name ‘Bossiesmans’ (Afrikaans for ‘Bushmen’) [5].

Despite ongoing debate on the use of ethnic labels, some recommendations are available [6] (Supplemental Web Resource S1). The Working Group of Indigenous Minorities in Southern Africa and the South African San Institute now represent the Indigenous communities in the region. Following the 2003 African Human Genome Initiative conference the two institutions declared a preference for their individual community names or collectively as San. The term Khoe–San was optional when discussing the San and Khoikhoi (more properly spelled as Khoekhoe) as a collective. ‘Khoisan’ was used by anthropologist Leonard Schultze to refer to the pastoralist Khoi and the hunter-gatherer San. As the term persists in linguistics for the broad and diverse language area, we chose to use it in our work (modified as ‘KhoeSan’). We do not assume that those included are linguistically, culturally or genetically homogenous.

The term Bantu means ‘people’ in the Bantu languages. While the term is not offensive across Africa, in Southern Africa the term can be seen as offensive because of its use by the Apartheid regime in South Africa. As such we opted for using the term solely in the linguistic context of referring to the collection of people who speak the Bantu languages, i.e. Bantu speaking communities. We use the following case structure for Bantu speaking community names and languages.
For Nguni communities, the prefix, which indicates noun class and plural/singular, is lower case followed by a capitalised root word (e.g., amaZulu and isiZulu).
For the other Bantu speaking communities, we use a leading capital letter for proper nouns (e.g. Sesotho and Basotho).

Lastly, as our paper attempts to investigate possible genetic distinctions between ‘vanished’ communities who are know mostly, if not entirely, from historic texts, we necessarily need to consider the terminology used in historic texts. Many of these terms have been identified by communities as derogatory and should be avoided [5, 6]. As we do not want to make the field more complicated by introducing new terms, we chose to use the term ‘Bushman’ but solely

to make it clear to which historic references we are discussing.

We are not referring to any contemporary people, participants or communities as ‘Bushmen’ nor do we consider the term ‘Bushman’ to be a legitimate way to refer to people except where it may be the preferred term (e.g. in the Kalahari).
Furthermore, our results show that the notion of a genetically distinct ‘Bushman’ community is unsupported and the term may have no value beyond discussing the use of the word in colonial literature.

Supplemental Note S2: Ethical concerns and data sharing.

Ethical concerns while working with the Baphuthi

Population genetic research necessarily uses information about the genetic identity of participants to discuss the history, present state or future of communities. It is therefore essential that there is a stable channel for communication and understanding between the community and the researchers directly involved in the work to avoid and/or mitigate any possible consequences of the research attention. The public release of genetic and genomic information further adds the need for clear and agreed upon general ethic principles to which researchers, ethics boards and academic journals can adhere and which can be discussed with the participants. With this work we have followed the guidance of the H3Africa working group for genetic research with African communities (Supplemental Web Resource S1). The working group developed a framework for the best practices and an interrogation of the concerns.

In population genetics, the collection of individual results will impact the discussion of and possibly the state of the community to which the individual belongs. The process then of liaising with communities as a collective and/or with community leaders as representatives of the collective is necessary. In our work, communities of interest were approached through Dr Sam Challis and Mr Puseletso Lecheko who have ongoing collaborations with the Baphuthi and neighbouring communities as part of the work of the Rock Art Research Institute. These existing collaborations and the expertise of Dr Challis and Mr Puseletso afforded us much trust with the communities. The community leaders (Chiefs) were informed of the project prior to sampling through in-person visits by our team. The proposed research was explained to the leader and we sought his approval for interviewing people of the community. When verbal approval was granted, we proceeded with the project. The community members who were interested in taking part in the study were provided the opportunity to discuss and question the details of the research with the researchers and with the assistance of a translator.

Data sharing

The data presented in this study was collected for the present study and ongoing work on the history and dynamics of the pre-colonial era for the Southern Bantu-speakers.
Ethics approval and participant written consent allows for the use of the data in future research by researchers not affiliated with the primary data collection team provided that the research abides by the agreement in the original consent forms, ethics application and is deemed appropriate by the primary data collection team. To avoid any misunderstandings, conflicts of interest and violations of the ethics agreement, the request for data sharing will conditioned upon signing a Data Transfer Agreement, supervised by the Technology Transfer Office and the Research Ethics Committee of the UWC, based on the principles stated in the signed Consent Form and information distributed to the participants.
We reserve the right to have the data withdrawn from any work where there has not been adequate discussion prior to the initiation of the project or where the objectives of the project are in conflict with the ethics approval and consent in the original data collection.

Our ethics approval allows for:
- The merging of the current data set with existing or newly generated data.
- The use of existing and/or new data analyses techniques on the data.
- Academic research work which is in a similar vein as the current project, i.e. population genomics, human history, genetic anthropology. We recognise that these are relatively broad areas thus it is necessary for researchers to contact the corresponding authors for data access.
- Further ethics approval applications at the University of the Western Cape, South Africa for work which is deemed to be a meaningful deviation from the original ethics approval and consent.

Our ethics approval does not allow for:
- Any work which directly uses the data for medical, gene – function or evolutionary selection research.
- Any attempt to use the individual genetic profiles to discuss individual phenotypes.
- Any attempt to individually identify the participants based on any of the data (genetic or other).

The provided data will be scrubbed of any identifying information but we further state that any attempt to approach/contact the individual participants based on the provided data for further data collection or to relate other sources of data to individual participants is not permitted.

Supplemental Note S3: Discussion of global PCA results

The first 5 PCs accounted for ~11% of the total variation. Principal component 1 (PC1, explaining ~6% of the variation) separated African from Eurasian individuals (Figure S1, Figure S2). Principal component 2 (~2% of the variation) separated eastern and western Eurasians (FigureS1, Figure S2). Along PC3 (2% of the variation) the KhoeSan individuals are separated from the non-KhoeSan Africans. Principal components 1 and 3 show the Baphuthi from Lesotho and South Africa plot close to the southern Bantu-speaking populations (e.g. amaZulu and Duma San). The Lake Chrissie San are distinctly closer to the Naro, Ju/'hoansi and G|ui and G||ana compared to Duma San and Baphuthi individuals. The recently admixed southern KhoeSan groups (‡Khomani, Nama and Karretjie) are spread toward Eurasian groups along PC1 which is not seen for the Baphuthi nor Lake Chrissie or Duma San.

An east African component found in the horn of Africa (Somali, Oromo, Amhara) is identified by PC4 (Figure S1). On this PC the Baphuthi, Duma San and southern Bantu-speaking groups are shifted toward the KhoeSan groups, away from east Africans. On PC5, west Africans are separated from east and southern Africans and here we see the Baphuthi and Duma San are at the extreme end of the Southern African cline, beyond the other SBE (Figure S1, Figure S2). The Lake Chrissie San are closer to the Hai||om than the Baphuthi and Duma San on PC5.

While accounting for <1% of the variation, PC 7 and 9 highlights two interesting affinities in the Baphuthi. On PC7 the Baphuthi plot at the extreme end near the Southern African KhoeSan, as opposed to the Juu and Khoe-Kwadi at the other end. The Southern African SBE are also shifted in this direction but the Baphuthi are well beyond other South African KhoeSan descendants (Duma San, Lake Chrissie San).
The PC9 distinguishes a western/Juu (e.g. Xun, Ju/'hoansi) KhoeSan component from a southern/Taa (‡Khomani, Karretjie) and Khoe-Kwadi component (Nama etc.) (Figure S1, Figure S2). While the Southern African SBE (including the Duma San) are shifted toward the western/Juu compared to the eastern African SBE, the Baphuthi from Lesotho are shifted further. The Lake Chrissie San, in contrast, are slightly off centre toward the southern/Taa groups.

The outlying position of the Baphuthi_LE along PC5 and 7 is unlikely due to a SNP-chip artefact as we do not see a unique Baphuthi_LE component in the unsupervised ADMIXTURE analyses (Figure S4). This suggests that the outlying position instead reflects possible another evolutionary cause within the Baphuthi related specifically to a reduction in diversity in the KhoeSan (PC9) and southern Bantu-speaker affinities (PC5).

2 Supplemental Figures

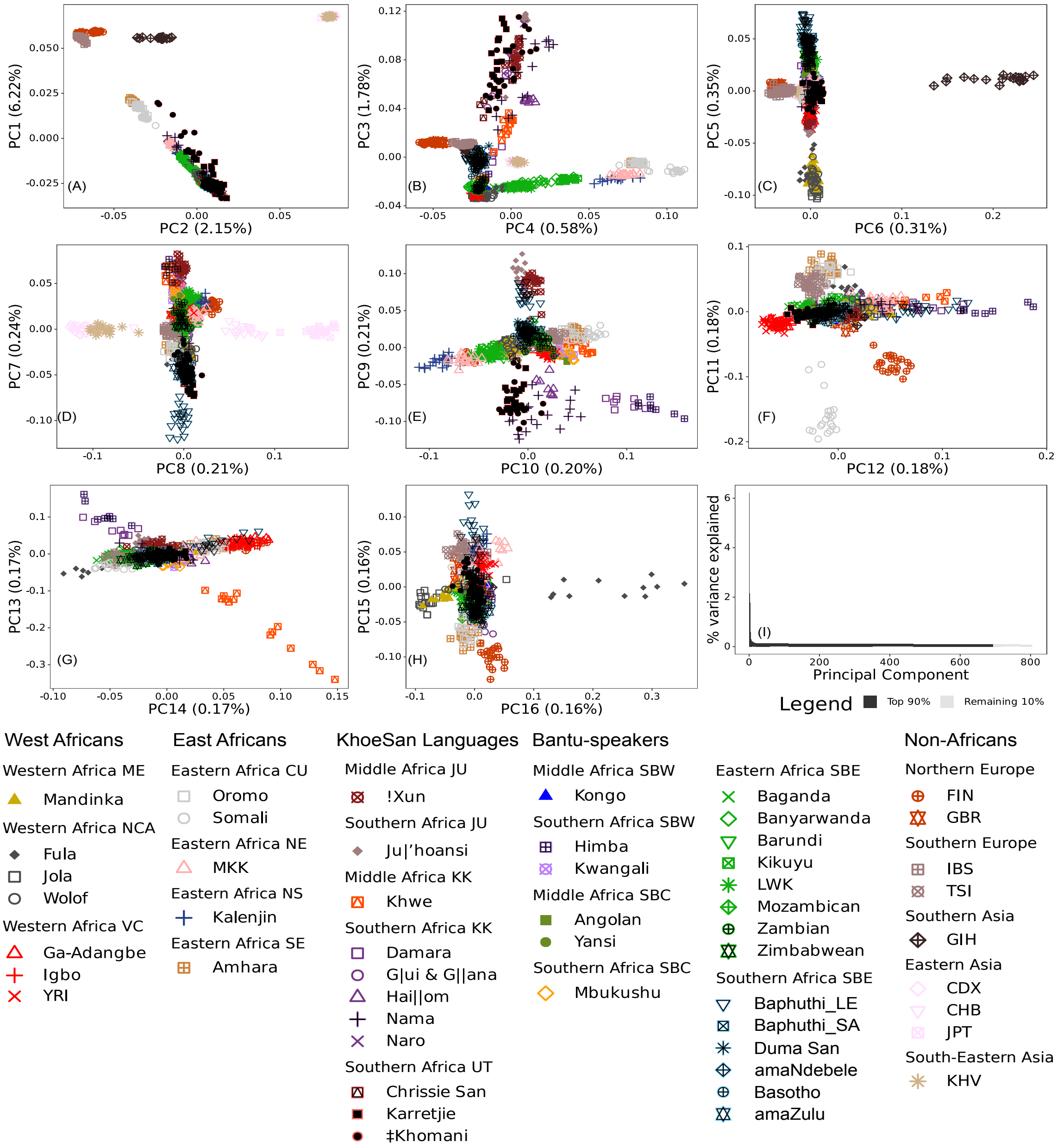

PC1 (6.22%)
PC2 (2.15%)
(A)
PC3 (1.78%)
PC4 (0.58%)
(B)
PC5 (0.35%)
PC6 (0.31%)
(C)
PC7 (0.24%)
PC8 (0.21%)
(D)
PC9 (0.21%)
PC10 (0.20%)
(E)
PC11 (0.18%)
PC12 (0.18%)
(F)
PC13 (0.17%)
PC14 (0.17%)
(G)
PC15 (0.16%)
PC16 (0.16%)
(H)
% variance explained
Principal Component
(I)
Legend
Top 90%
Remaining 10%
West Africans
Western Africa ME
Mandinka
Western Africa NCA
Fula
Jola
Wolof
Western Africa VC
Ga-Adangbe
Igbo
YRI
East Africans
Eastern Africa CU
Oromo
Somali
Eastern Africa NE
MKK
Eastern Africa NS
Kalenjin
Eastern Africa SE
Amhara
KhoeSan Languages
Middle Africa JU
!Xun
Southern Africa JU
Ju|'hoansi
Middle Africa KK
Khwe
Southern Africa KK
Damara
G|ui & G||ana
Hai||om
Nama
Naro
Southern Africa UT
Chrissie San
Karretjie
‡Khomani
Bantu-speakers
Middle Africa SBW
Kongo
Southern Africa SBW
Himba
Kwangali
Middle Africa SBC
Angolan
Yansi
Southern Africa SBC
Mbukushu
Eastern Africa SBE
Baganda
Banyarwanda
Barundi
Kikuyu
LWK
Mozambican
Zambian
Zimbabwean
Southern Africa SBE
Baphuthi_LE
Baphuthi_SA
Duma San
amaNdebele
Basotho
amaZulu
Non-Africans
Northern Europe
FIN
GBR
Southern Europe
IBS
TSI
Southern Asia
GIH
Eastern Asia
CDX
CHB
JPT
South-Eastern Asia
KHV

Figure S1: Principal component analysis of all data. PCA plots in subplots A – H and histogram of percentage variation explained by each component shown in subplot I. Focal populations are indicated in the plots by a black symbol overlaying the coloured symbol. Colours indicate regional and linguistic divisions. Linguistic abbreviations: Mande - ME, North-Central Atlantic - NCA, Volta-Congo - VC, Cushtic – CU, Nilotic eastern - NE, Nilotic southern- NS, Semitic - SE, southern Bantoid western Bantu– SBW, southern Bantoid central western Bantu– SBC, southern Bantoid eastern Bantu– SBE, Juu KhoeSan – JU, Khoe-Kwadi KhoeSan – KK, Ui! and Taa KhoeSan – UT.

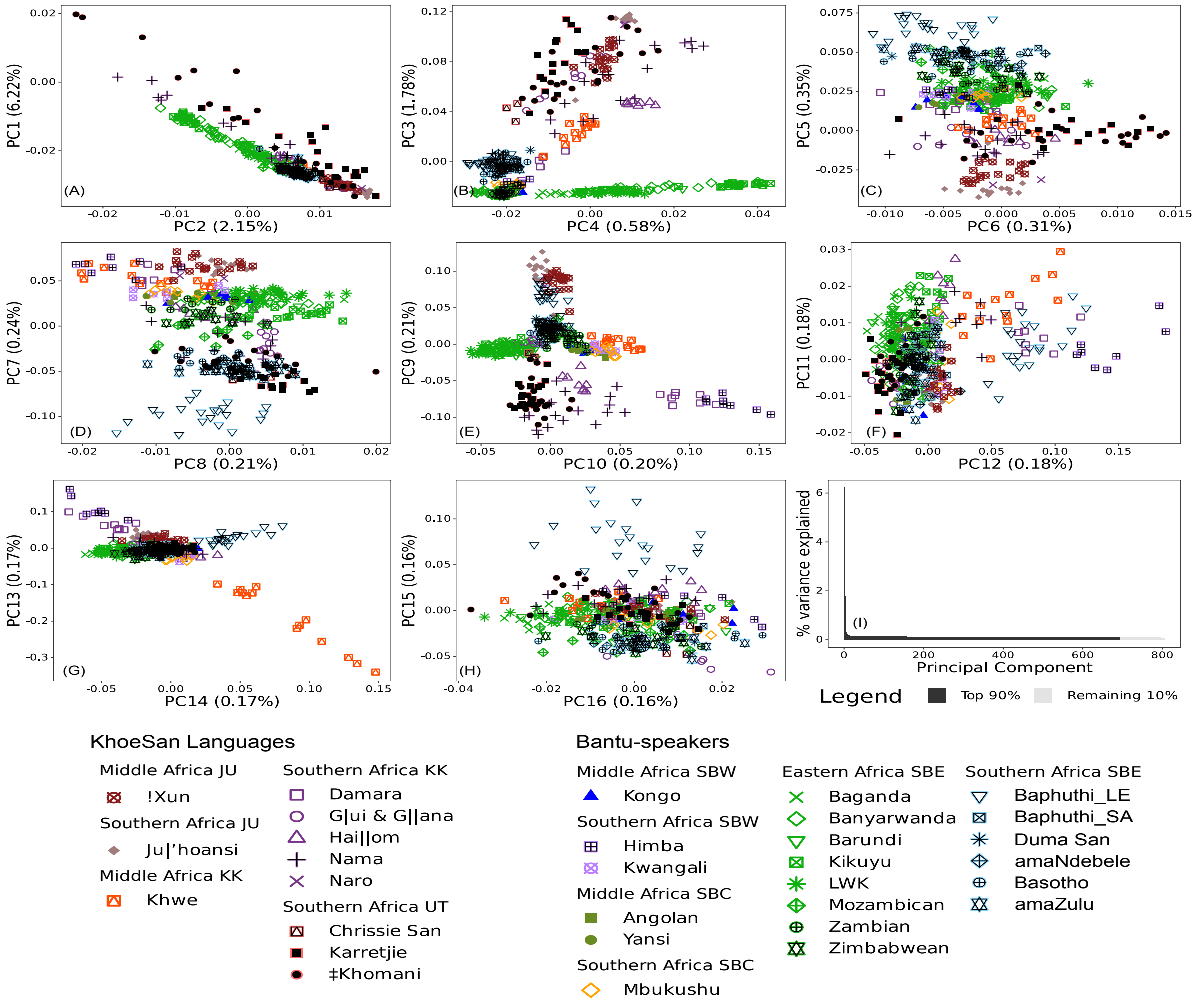

Figure S2: Principal component analysis of Southern African data. PCA plots in subplots A – H and histogram of percentage variation explained by each component shown in subplot I. Focal populations are indicated in the plots by a black symbol overlaying the coloured symbol. Colours indicate regional and linguistic divisions. Linguistic abbreviations: Mande - ME, North-Central Atlantic - NCA, Volta-Congo - VC, Cushtic – CU, Nilotic eastern - NE, Nilotic southern- NS, Semitic - SE, southern Bantoid western Bantu– SBW, southern Bantoid central western Bantu– SBC, southern Bantoid eastern Bantu– SBE, Juu KhoeSan – JU, Khoe-Kwadi KhoeSan – KK, Ui! and Taa KhoeSan – UT.

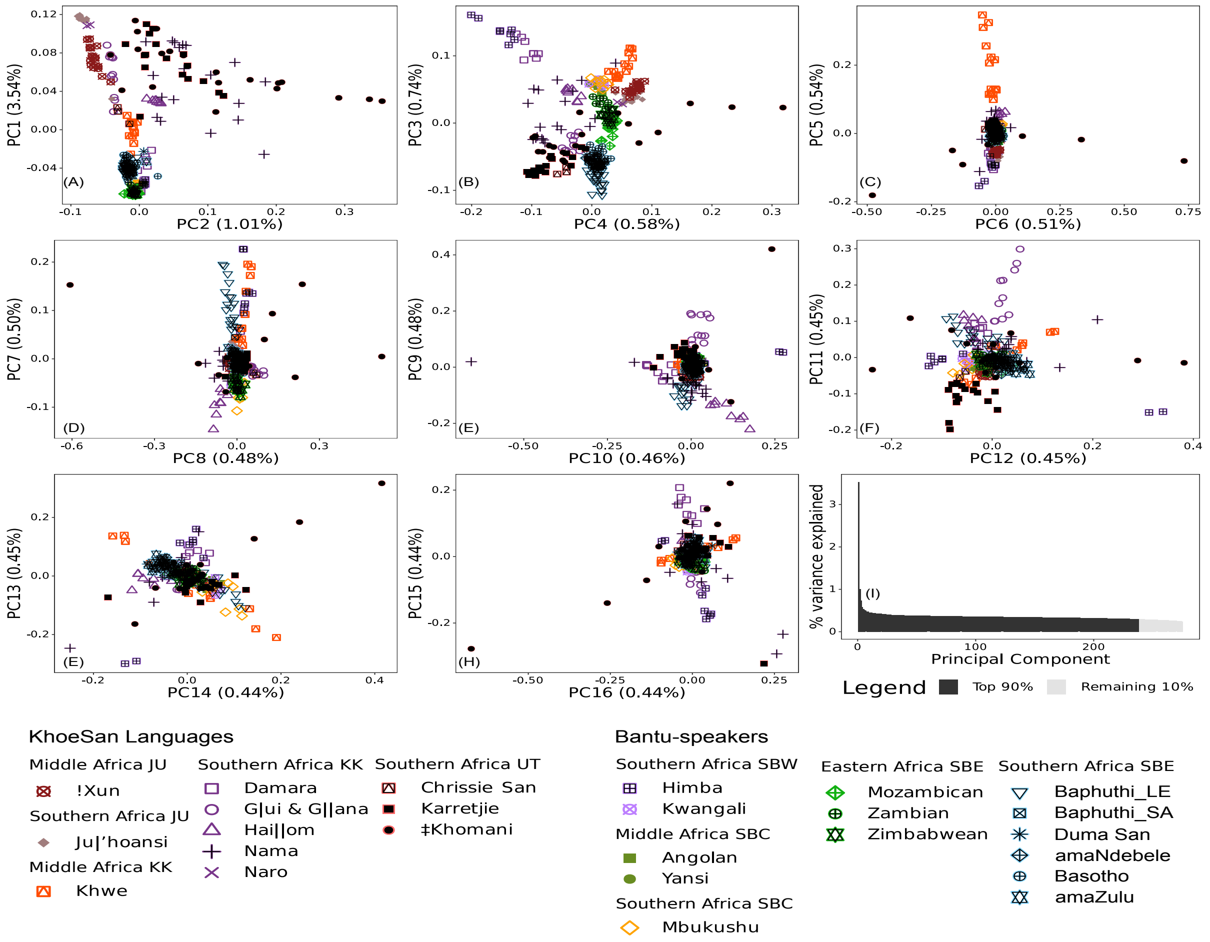

Figure S3: Principal component analysis of all data, plotting only the Southern African samples. PCA plots in subplots A – H and histogram of percentage variation explained by each component shown in subplot I. Focal populations are indicated in the plots by a black symbol overlaying the coloured symbol. Colours indicate regional and linguistic divisions. Linguistic abbreviations: Mande - ME, North-Central Atlantic - NCA, Volta-Congo - VC, Cushtic – CU, Nilotic eastern - NE, Nilotic southern- NS, Semitic - SE, southern Bantoid western Bantu– SBW, southern Bantoid central western Bantu– SBC, southern Bantoid eastern Bantu– SBE, Juu KhoeSan – JU, Khoe-Kwadi KhoeSan – KK, !Ui and Taa KhoeSan – UT.

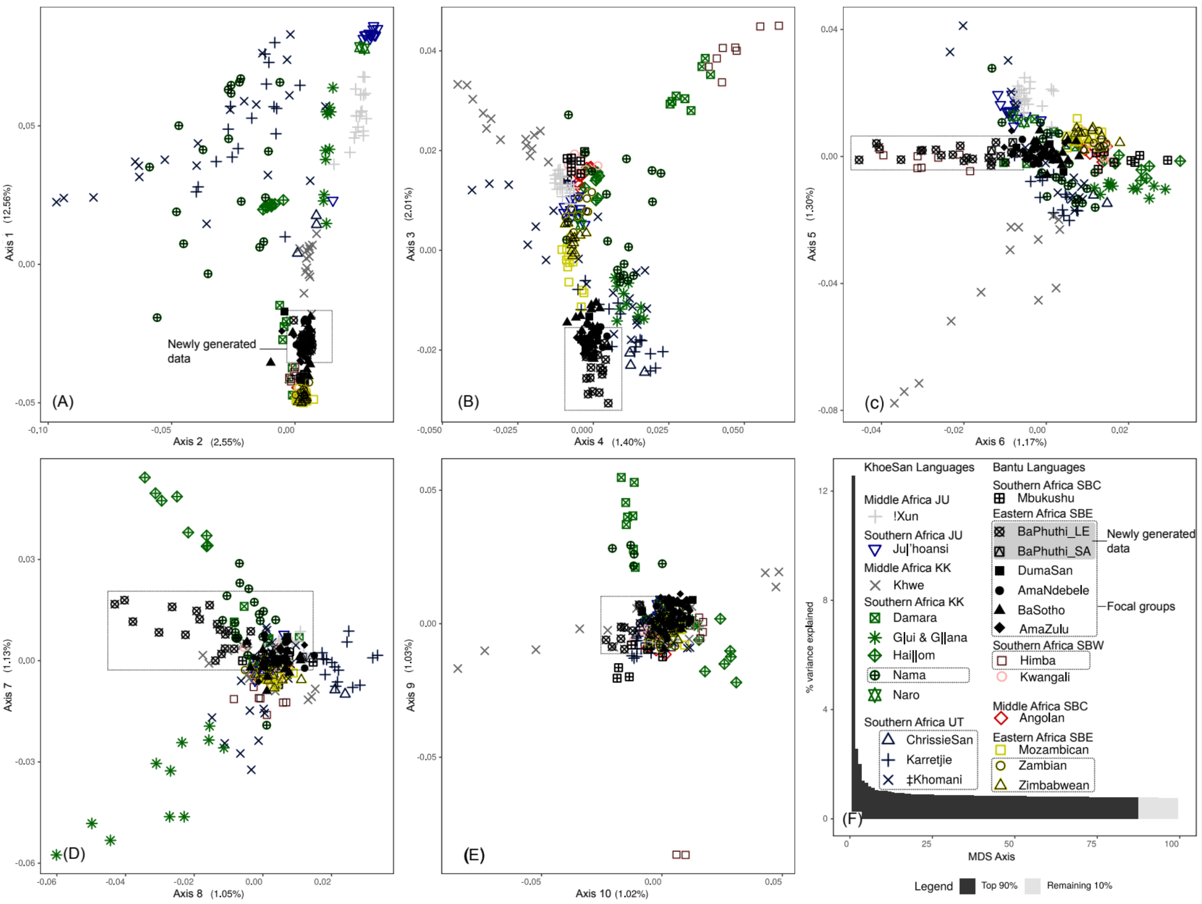

Figure S4: Multidimensional scaling analysis of only the Southern African samples. Focal populations are indicated in the plots by a black symbol overlaying the coloured symbol and by dotted boxes in the figure key. Scaling plots in subplots A – E and histogram of percentage variation explained by each component shown in subplot F. Colours indicate the regional and linguistic divisions. The newly generated data are indicated with a dotted box in both the figure key and the plot. Linguistic abbreviations: Mande - ME, North-Central Atlantic - NCA, Volta-Congo - VC, Cushtic – CU, Nilotic eastern - NE, Nilotic southern- NS, Semitic - SE, southern Bantoid western Bantu– SBW, southern Bantoid central western Bantu– SBC, southern Bantoid eastern Bantu– SBE, Juu KhoeSan – JU, Khoe-Kwadi KhoeSan – KK, !Ui and Taa KhoeSan – UT.

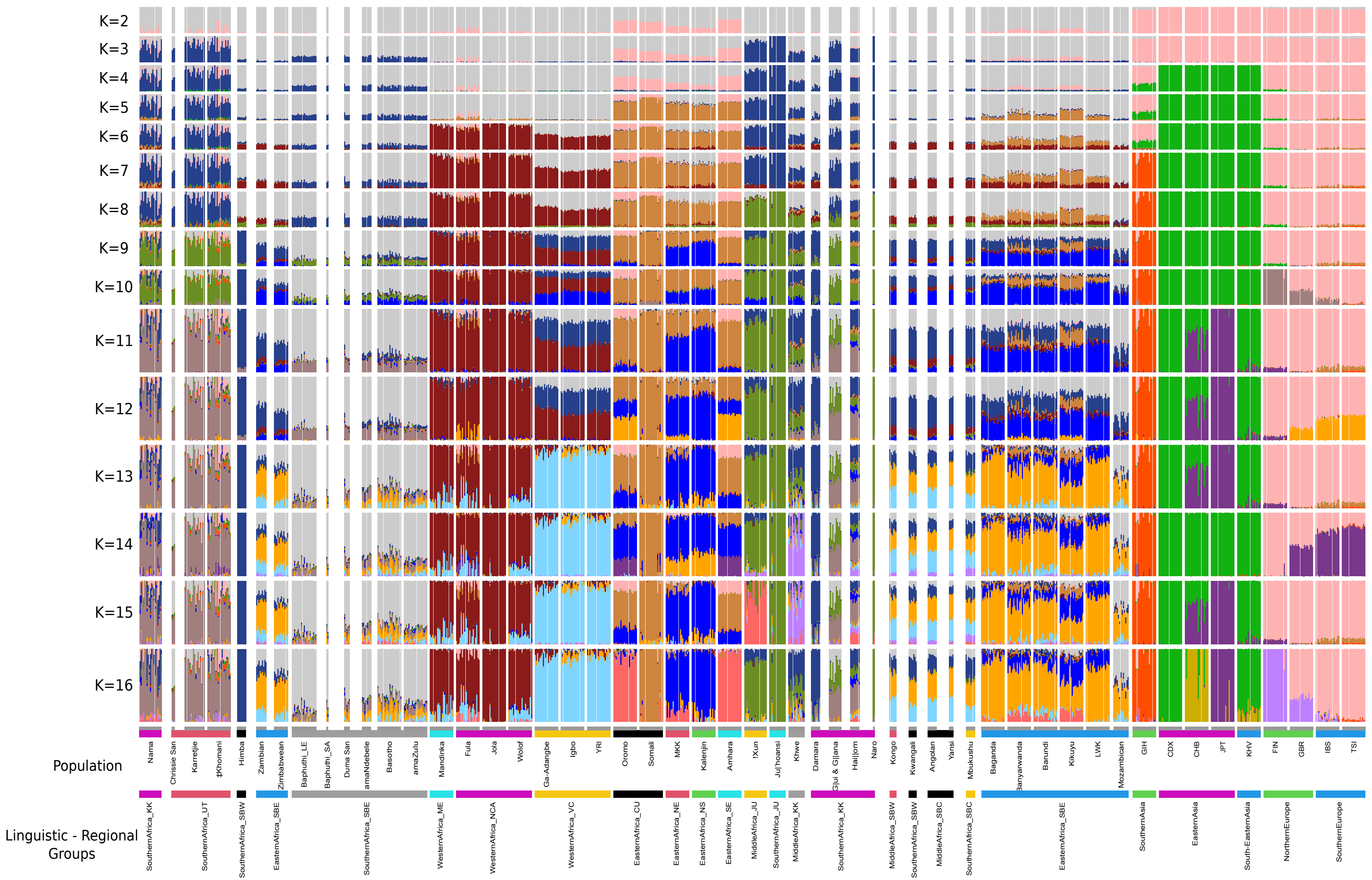

K=2
K=3
K=4
K=5
K=6
K=7
K=8
K=9
K=10
K=11
K=12
K=13
K=14
K=15
K=16
Population
Nama
Chrissie San
Karretjie
‡Khomani
Himba
Zambian
Zimbabwean
Baphuthi_LE
Baphuthi_SA
Duma San
amaNdebele
Basotho
amaZulu
Mandinka
Fula
Jola
Wolof
Ga-Adangbe
Igbo
YRI
Oromo
Somali
MKK
Kalenjin
Amhara
!Xun
Ju|'hoansi
Khwe
Damara
Gui & Gllana
Hai||om
Naro
Kongo
Kwangali
Angolan
Yansi
Mbukushu
Baganda
Banyarwanda
Barundi
Kikuyu
LWK
Mozambican
GIH
CDX
CHB
JPT
KHV
FIN
GBR
IBS
TSI
Linguistic - Regional Groups
SouthernAfrica_KK
SouthernAfrica_UT
SouthernAfrica_SBW
EasternAfrica_SBE
SouthernAfrica_SBE
WesternAfrica_ME
WesternAfrica_NCA
WesternAfrica_VC
EasternAfrica_CU
EasternAfrica_NE
EasternAfrica_NS
EasternAfrica_SE
MiddleAfrica_JU
SouthernAfrica_JU
MiddleAfrica_KK
SouthernAfrica_KK
MiddleAfrica_SBW
SouthernAfrica_SBW
MiddleAfrica_SBC
SouthernAfrica_SBC
EasternAfrica_SBE
SouthernAsia
EasternAsia
South-EasternAsia
NorthernEurope
SouthernEurope

Figure S5: ADMIXTURE proportions for K=2 ...16 represented as a stacked bar graph for each individual. Each colour represents a component. Samples have been grouped by Linguistic-geographic regions. Population abbreviations are explained in Table S1.

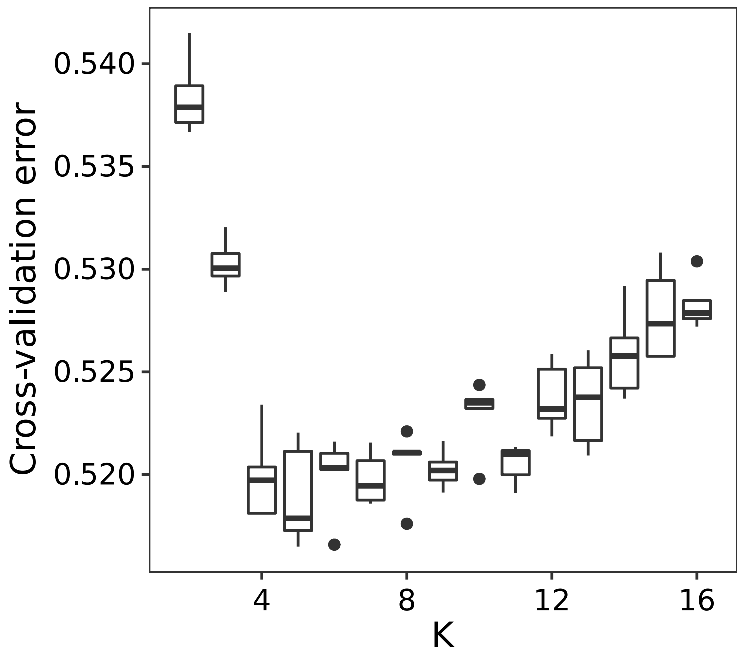

Figure S6: Changes in cross-validation error for K=2..16 with 10 repeated runs each. Dots indicate outlying values. Median value indicated by the line within each box, and the interquartile range is indicated by the extent of the boxes.

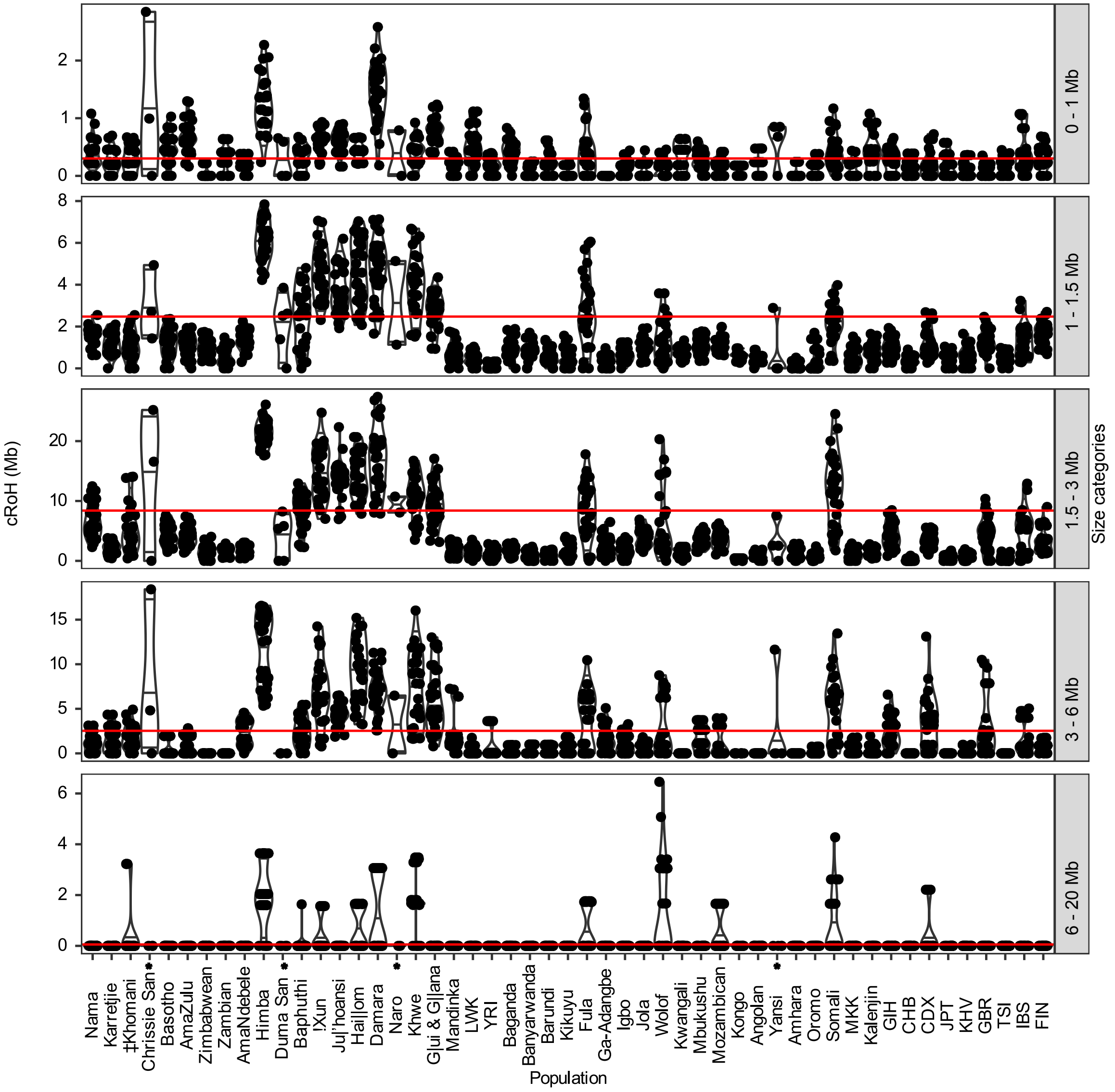

cRoH (Mb)
Size categories
0 - 1 Mb
1 - 1.5 Mb
1.5 - 3 Mb
3 - 6 Mb
6 - 20 Mb
Population
Nama
Karretjie
‡Khomani
Chrissie San*
Basotho
AmaZulu
Zimbabwean
Zambian
AmaNdebele
Himba
Duma San*
Baphuthi
!Xun
Ju|'hoansi
Hai||om
Damara
Naro*
Khwe
G|ui & G||ana
Mandinka
LWK
YRI
Baganda
Banyarwanda
Barundi
Kikuyu
Fula
Ga-Adangbe
Igbo
Jola
Wolof
Kwangali
Mbukushu
Mozambican
Kongo
Angolan
Yansi*
Amhara
Oromo
Somali
MKK
Kalenjin
GIH
CHB
CDX
JPT
KHV
GBR
TSI
IBS
FIN

Figure S7: Cumulative runs of homozygosity (cRoH) for each population across a set of five RoH size bins. Size bins are indicated on the right. Individual dots indicate the average from four individuals in a single iteration (a total of 30 iterations per population). In populations marked with an * we show the estimates for individuals, not iterations, due to small sample sizes. The red line shows the mean of the Baphuthi. Horizontal lines in each violin indicates the 25%, 50% and 75% quantiles.

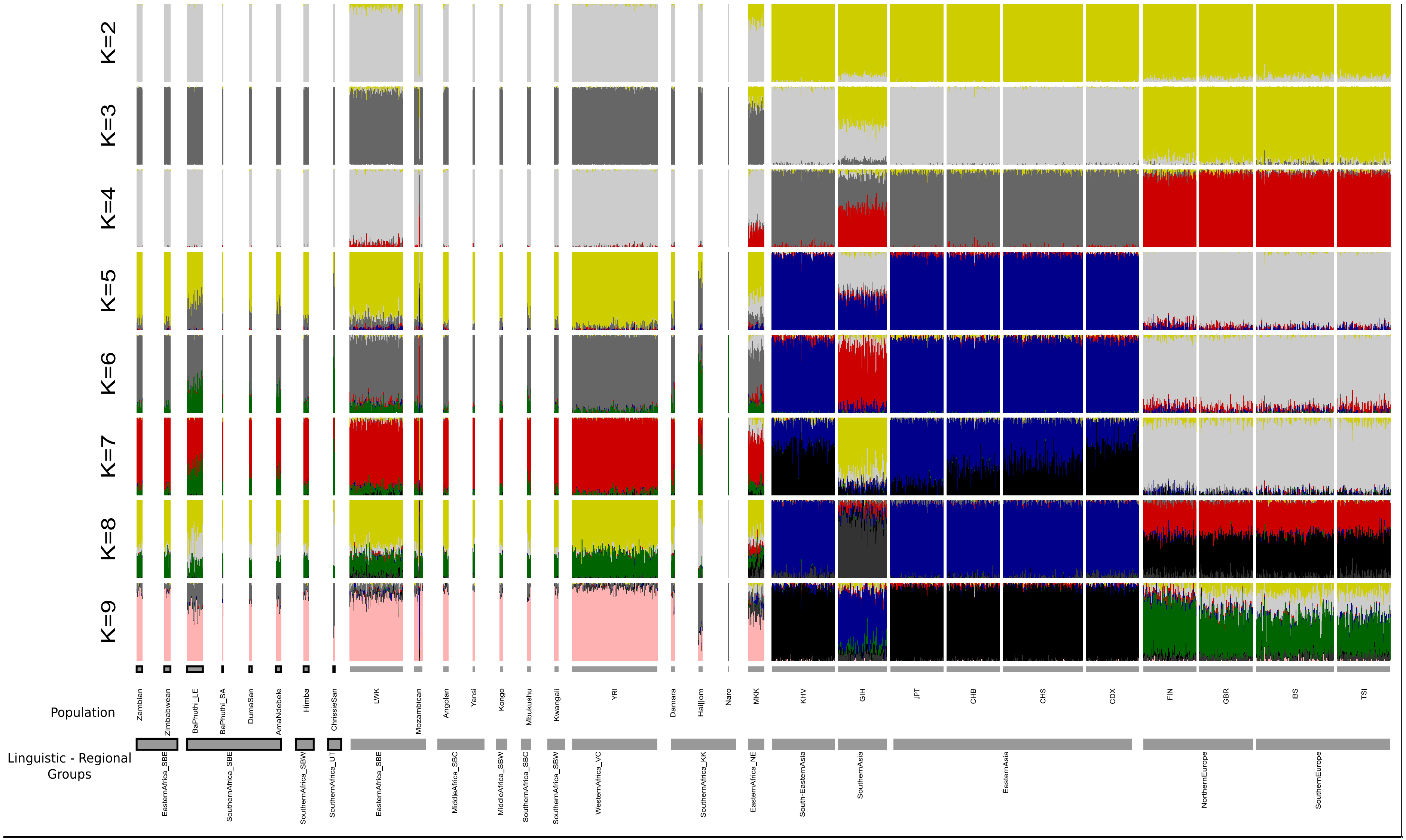

Figure S 8: ADMIXTURE proportions based on the X chromosome data for K=2 ...9 represented as a stacked bar graph for each individual. Each colour represents a component. Samples have been grouped by Linguistic-geographic regions (bars along the x axis). Population abbreviations are explained in Table S1. Focal populations indicated in x axis labels by black border around the bars.

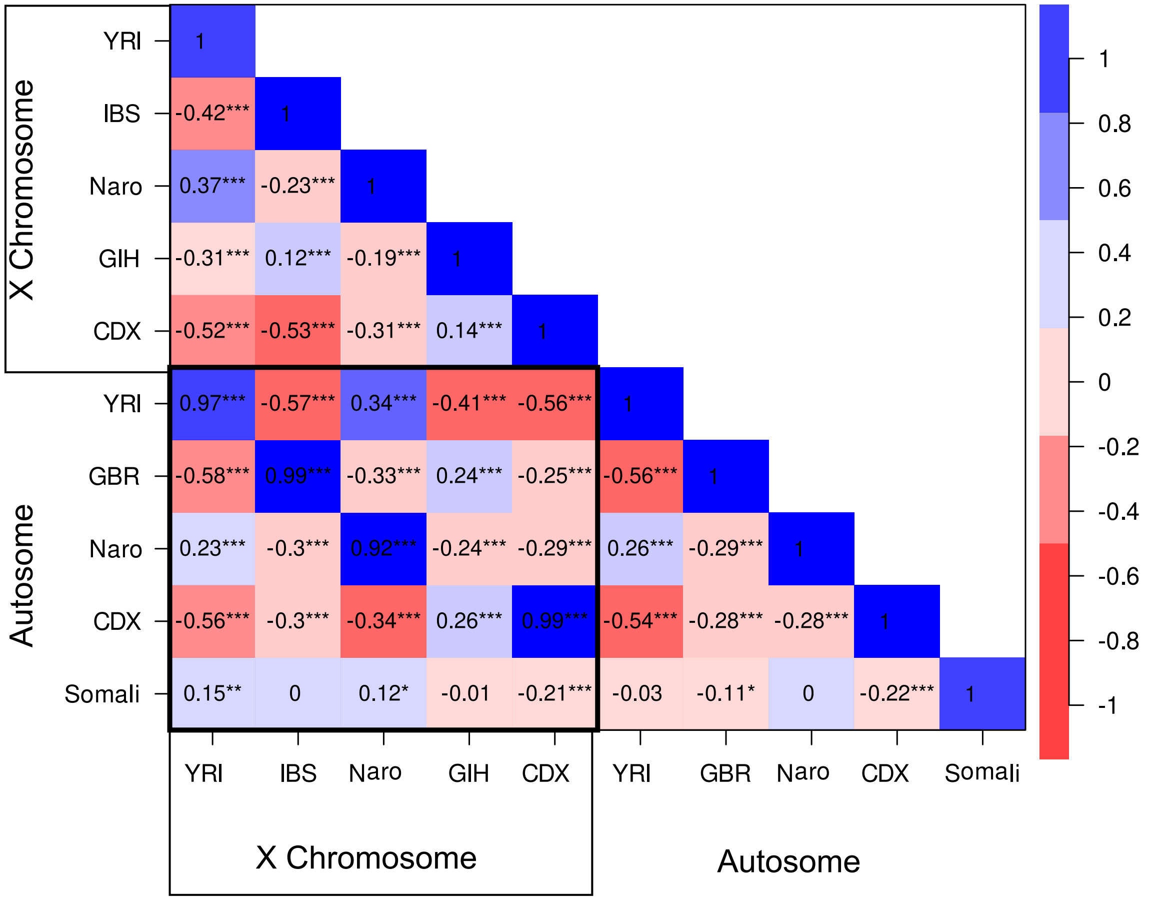

Figure S 9: Pearson's correlation coefficients estimated between pairs of ADMIXTURE components from the autosomal and X chromosome data. Colours in the plot and scale bar correspond to the R-squared value and asterisk indicate level of significance for the p-values. * < 0.05, ** < 0.01, *** < 0.001. Population labels along the x and y axes correspond to the population in which the component was at its greatest, i.e. the proxy source for the ancestry.

2.1 Supplemental Tables

Table S1: List of data included in this paper. Sample sizes after quality control steps (n) are indicated. Language information taken from Glottolog v4 (Web Resource S2). The PubMed ID (PMID) is provided for each data source.

Table S2: Pairwise post-hoc test results for significant differences between populations when comparing ADMIXTURE components at K=9. In the matrix of p-values, the upper right are Nemenyi post-hoc results and the lower left are Baumgartner-Weiß-Schindler results. Statistically significant p-values indicated in *italics*.

Table S3: Estimates of the ADMIXTURE components in each population at K = 9. Indicated are the mean (μ), minimum (Min), maximum (Max) and standard deviation (s.d.) within each population for each component and for selected combinations of components.

Table S4: Results of formal test for admixture using f_3 estimates.

Table S5: Admixture date estimates considering pairs of sources using linkage disequilibrium decay curves. Detected events have been grouped by the language and regions of the identified sources and the date estimates. Where multiple events were detected, the number of events is indicated under 'event'. Symbols : μ – Mean, s.d. – Standard deviation.

Table S6: Estimates of the median ADMIXTURE components in each population for autosomal and X chromosome at K = 5. Indicated are the means within populations for each component (Autosomal and X chromosome) and the ratio of Autosomal:X chromosome for each component. Ratio values were capped at 30 and 1e-06 was added to all components to avoid ratios with a 0 numerator or denominator.

Supplemental Web Resources

Web Resource S1:
H3 Africa Working Group Report 2011. Harnessing Genomic Technologies Toward Improving Health in Africa : Opportunities and Challenges. http://h3africa.org/About/White-Paper

Web Resource S2:
Hammarström, H., Forkel, R. and Haspelmath, M. (2019). Glottolog 4.0. https://glottolog.org/

Supplemental References

1. Berg, K., Bonham, V., Boyer, J., Brody, L., Brooks, L., Collins, F., Guttmacher, A., McEwen, J., Muenke, M., Olson, S. et al. (2005). The Use of Racial, Ethnic, and Ancestral Categories in Human Genetics Research. Am. J. Hum. Genet. *77*, pp. 519-532.
2. Tang, H., Quertermous, T., Rodriguez, B., Kardia, S.L.R., Zhu, X., Brown, A., Pankow, J.S., Province, M.A., Hunt, S.C., Boerwinkle, E. et al. (2005). Genetic Structure, Self-Identified Race/Ethnicity, and Confounding in Case-Control Association Studies. Am. J. Hum. Genet. *76*, pp. 268-275.
3. Morning, A. (2014). And you thought we had moved beyond all that: biological race returns to the social sciences. Ethnic Racial Stud. *37*, pp. 1676-1685.
4. Kent, S. (2002). Interethnic Encounters of the First Kind: An Introduction. In Ethnicity, Hunter-Gatherers, and the "Other": Association or Assimilation in Africa, Kent, S. ed. (Smithsonian Institution Press).
5. Schlebusch, C.M. (2010). Issues raised by use of ethnic-group names in genome study. Nature *464*, pp. 487.

6. Chennells, R. and Steenkamp, A. (2016). International Genomics Research involving the San People. In “Ethics Dumping” – Paradigmatic Case Studies: A report for TRUST, Schroeder, D., Lucas, J. C., Fenet, S. and Hirsch, F. eds. (TRUST Project), pp. 35-40.
